# Altered longitudinal structural connectome in paediatric mild traumatic brain injury: an Advancing Concussion Assessment in Paediatrics study

**DOI:** 10.1093/braincomms/fcad173

**Published:** 2023-05-31

**Authors:** Ashley L Ware, Adrian I Onicas, Nishard Abdeen, Miriam H Beauchamp, Christian Beaulieu, Bruce H Bjornson, William Craig, Mathieu Dehaes, Sylvain Deschenes, Quynh Doan, Stephen B Freedman, Bradley G Goodyear, Jocelyn Gravel, Andrée-Anne Ledoux, Roger Zemek, Keith Owen Yeates, Catherine Lebel

**Affiliations:** Department of Psychology, Georgia State University, Atlanta, GA 30303, USA; Department of Psychology, University of Calgary, Calgary, AB T2N 0V2, Canada; Alberta Children's Hospital Research Institute and Hotchkiss Brain Institute, University of Calgary, AB T2N 0V2, Canada; Department of Psychology, University of Calgary, Calgary, AB T2N 0V2, Canada; Computer Vision Group, Sano Centre for Computational Medicine, Kraków 30-054, Poland; Department of Radiology, Children’s Hospital of Eastern Ontario Research Institute, University of Ottawa, Ottawa, ON, Canada K1H 8L1; Department of Psychology, University of Montreal and CHU Sainte-Justine Hospital Research Center, Montréal, QC, Canada H3C 3J7; Department of Biomedical Engineering, 1098 Research Transition Facility, University of Alberta, Edmonton, AB, Canada T6G 2V2; Division of Neurology, Department of Pediatrics, University of British Columbia, Vancouver, BC, Canada V6H 3V4; BC Children’s Hospital Research Institute, Vancouver, BC, Canada V6H 3V4; University of Alberta and Stollery Children’s Hospital, Edmonton, AB, Canada T6G 1C9; Department of Radiology, Radio-oncology and Nuclear Medicine, Institute of Biomedical Engineering, University of Montreal, Montréal, QC, Canada H3T1J4; CHU Sainte-Justine Research Center, Montréal, QC, Canada H3T1C5; CHU Sainte-Justine Research Center, Montréal, QC, Canada H3T1C5; Department of Radiology, Radio-oncology and Nuclear Medicine, University of Montreal, Montréal, QC, CHU Sainte-Justine Research Center, Montréal, QC, Canada H3T1C5; Department of Pediatrics University of British Columbia, BC Children’s Hospital Research Institute, Vancouver, BC, Canada V5Z 4H4; Departments of Pediatric and Emergency Medicine, Cumming School of Medicine, University of Calgary, Calgary, AB, Canada T3B 6A8; Alberta Children's Hospital Research Institute and Hotchkiss Brain Institute, University of Calgary, AB T2N 0V2, Canada; Department of Radiology, University of Calgary, Calgary, AB T2N 0V2, Canada; Pediatric Emergency Department, CHU Sainte-Justine, Montréal, QC H3T1C5, Canada; Department of Pediatric, Université de Montréal, Montréal, QC H3T 1C5, Canada; Department of Cellular Molecular Medicine, University of Ottawa, Children’s Hospital of Eastern Ontario Research Institute, Ottawa, ON, Canada K1H8L1; Department of Pediatrics and Emergency Medicine, University of Ottawa, Children’s Hospital of Eastern Ontario Research Institute, Ottawa, ON, Canada K1H8L1; Department of Psychology, University of Calgary, Calgary, AB T2N 0V2, Canada; Alberta Children's Hospital Research Institute and Hotchkiss Brain Institute, University of Calgary, AB T2N 0V2, Canada; Alberta Children's Hospital Research Institute and Hotchkiss Brain Institute, University of Calgary, AB T2N 0V2, Canada; Department of Radiology, University of Calgary, Calgary, AB T2N 0V2, Canada

**Keywords:** paediatric mild TBI, orthopaedic injury, structural connectome, graph theory, diffusion MRI

## Abstract

Advanced diffusion-weighted imaging techniques have increased understanding of the neuropathology of paediatric mild traumatic brain injury (i.e. concussion). Most studies have examined discrete white-matter pathways, which may not capture the characteristically subtle, diffuse and heterogenous effects of paediatric concussion on brain microstructure. This study compared the structural connectome of children with concussion to those with mild orthopaedic injury to determine whether network metrics and their trajectories across time post-injury differentiate paediatric concussion from mild traumatic injury more generally. Data were drawn from of a large study of outcomes in paediatric concussion. Children aged 8–16.99 years were recruited from five paediatric emergency departments within 48 h of sustaining a concussion (*n* = 360; 56% male) or mild orthopaedic injury (*n* = 196; 62% male). A reliable change score was used to classify children with concussion into two groups: concussion with or without persistent symptoms. Children completed 3 T MRI at post-acute (2–33 days) and/or chronic (3 or 6 months, via random assignment) post-injury follow-ups. Diffusion-weighted images were used to calculate the diffusion tensor, conduct deterministic whole-brain fibre tractography and compute connectivity matrices in native (diffusion) space for 90 supratentorial regions. Weighted adjacency matrices were constructed using average fractional anisotropy and used to calculate global and local (regional) graph theory metrics. Linear mixed effects modelling was performed to compare groups, correcting for multiple comparisons. Groups did not differ in global network metrics. However, the clustering coefficient, betweenness centrality and efficiency of the insula, cingulate, parietal, occipital and subcortical regions differed among groups, with differences moderated by time (days) post-injury, biological sex and age at time of injury. Post-acute differences were minimal, whereas more robust alterations emerged at 3 and especially 6 months in children with concussion with persistent symptoms, albeit differently by sex and age. In the largest neuroimaging study to date, post-acute regional network metrics distinguished concussion from mild orthopaedic injury and predicted symptom recovery 1-month post-injury. Regional network parameters alterations were more robust and widespread at chronic timepoints than post-acutely after concussion. Results suggest that increased regional and local subnetwork segregation (modularity) and inefficiency occurs across time after concussion, emerging after post-concussive symptom resolve in most children. These differences persist up to 6 months after concussion, especially in children who showed persistent symptoms. While prognostic, the small to modest effect size of group differences and the moderating effects of sex likely would preclude effective clinical application in individual patients.

## Teaser

Increased regional and local subnetwork segregation (modularity) and inefficiency occurred across time after concussion, emerging after post-concussive symptoms resolved in most children and persisted up to 6 months after mild traumatic brain injury, especially in children with persistent symptoms 1-month post-injury.

## Graphical Abstract

**Graphical Abstract:**
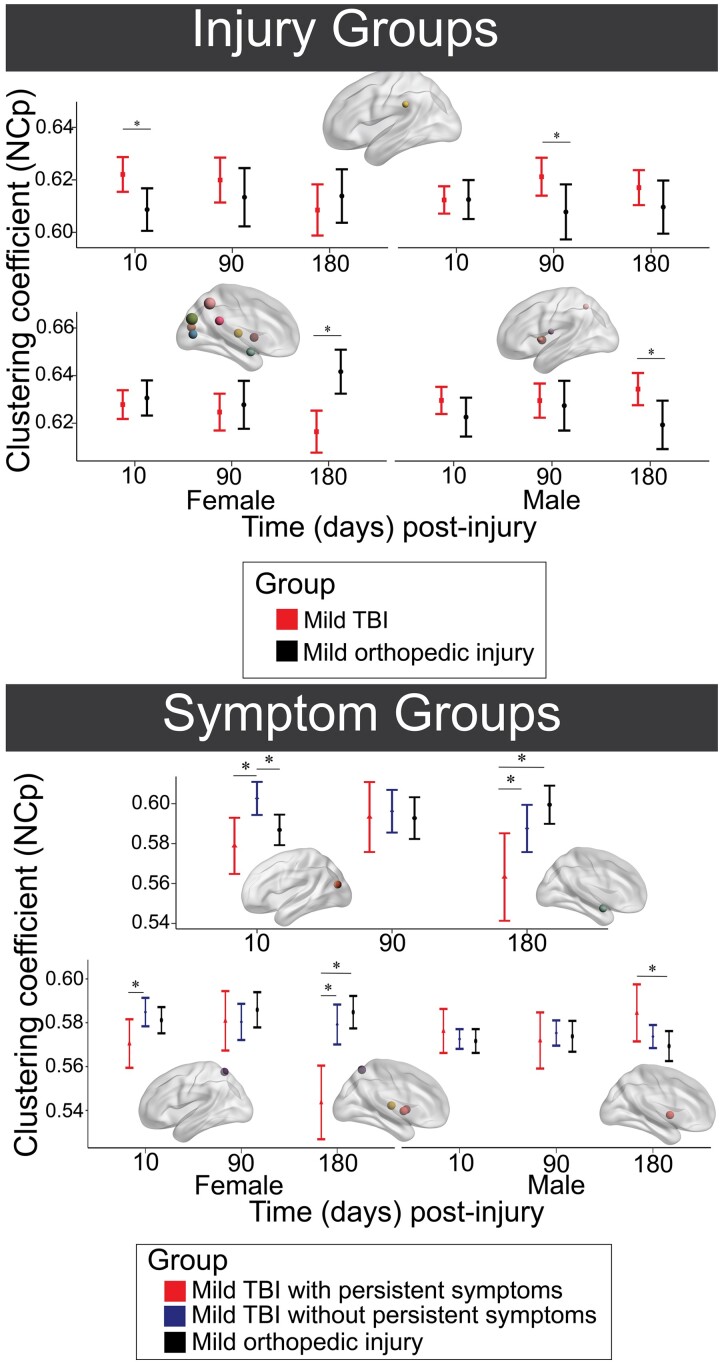

## Introduction

Each year, clinicians around the world struggle to identify mild traumatic brain injury (TBI) in children and predict whether children will demonstrate persistent symptoms and related functional impairment.^1^ Paediatric TBI is a major global public health concern that affects millions of children in North America annually, resulting in dramatic medical and societal costs.^2^ Most injuries (85–90%) are classified as mild in severity (i.e. concussion).^1,2^ In most cases, the post-concussive symptoms that commonly occur are most severe acutely, with recovery expected within several weeks post-injury. However, as many as 35% of injured children fail to recover within 1-month post-injury and experience persistent physical (e.g. post-traumatic headache), cognitive (e.g. reduced concentration) and emotional (e.g. irritability) complaints.^3,4^ Though urgently needed for clinical management, no objective clinical test or biomarker currently exists for accurately detecting mild TBI or predicting risk for delayed recovery in injured children.^5^

Paediatric mild TBI results in subtle alterations in widespread, heterogeneous brain regions.^6,7^ In contrast to more severe TBI, mild injury does not result in visible abnormalities on conventional clinical neuroimaging in the vast majority of children.^8^ The subtle effects of mild TBI are difficult to detect through standard neuroimaging, whether for detection of mild TBI or prognostication of its outcomes.^6^ Thus, the underlying neuropathology of mild TBI is unclear.^6,9–13^ Diffusion MRI techniques permit non-invasive, *in vivo* investigation of brain structure and have demonstrated high sensitivity to the subtle white-matter alterations associated with paediatric mild TBI in both early and late stages.^6,10,14,15^ Furthermore, advanced diffusion MRI metrics can predict clinical outcomes in paediatric mild TBI.^6,11,16^ Advanced diffusion MRI techniques may yield an objective, non-invasive biomarker of paediatric mild TBI.

Despite the promise of diffusion MRI techniques, most research on paediatric mild TBI has investigated isolated brain regions using tractography or voxel-wise comparisons.^17–20^ This limits understanding of the neurobiological effects of mild TBI because global brain processes occur through white-matter connections among distinct local networks comprised of specialized brain regions.^21–23^ The diffuse, microscopic alterations characteristic of mild TBI likely disrupt connectivity both within and between these specialized brain networks, culminating in the largely non-focal symptoms of mild TBI.^6,24,25^ This may explain the ineffectiveness of studying distinct brain regions in isolation for identification and prognostication of paediatric mild TBI.^19^ Structural connectomics applies graph theory and advanced diffusion MRI to examine white-matter connections among distributed brain regions. In contrast to isolated tracts, a sophisticated network-based approach, such as structural connectomics, may be able to elucidate a biomarker of mild TBI that is both sensitive and specific enough to the effects of mild TBI for clinical use.^24–28^

The structural connectome is poorly understood in paediatric mild TBI.^29^ Published studies are scarce to date and most have small sample sizes (*N* = 24) with restricted age ranges (e.g. age 11–16 years),^30^ biased sex ratios (e.g. 91% male),^30^ and variable methodologies, including different diagnostic criteria, time periods post-injury, comparison groups (e.g. children who are uninjured, typically developing^31^ or with an extracranial injury) and network construction approaches.^29–32^ Within the context of those limitations, mounting evidence suggests that paediatric mild TBI is associated with a more segregated and inefficient network topology.^29–33^ Global (e.g. greater clustering coefficient, small-worldness, characteristic path length and modularity, with reduced global efficiency) and regional (nodal; e.g. reduced nodal degree and clustering coefficient in several cortical regions) network alterations have been detected within 96 h following mild TBI.^30,31^ Changes in network structure can also be dynamic across time post-injury.^31,34^ Initial evidence from two longitudinal studies suggests that early alterations normalize within the first year post-injury and may benefit from clinical intervention (i.e. 6 weeks of aerobic exercise),^31,34^ although alterations in other network metrics can emerge at later periods,^31^ suggesting that the detection and trajectory of network structural changes may be feature (metric) dependent.

Three independent studies underscore the need to consider comparison group selection in paediatric mild TBI research. Previously, we contrasted two comparison groups to delineate the specific effects of mild TBI during childhood: a group of uninjured, typically developing children and a group of children with mild orthopaedic injury (OI).^35^ Features of the post-acute (i.e. ∼2 weeks post-injury) structural connectome were similar at both the global and regional levels of brain networks after mild TBI and mild OI, but differed between both injury groups and uninjured, typically developing children. Thus, network alterations were associated with mild traumatic injury more generally, regardless of whether the injury involved head trauma (i.e. involved the brain). Two additional studies of mild-to-severe paediatric TBI relative to extracranial injury^29^ and uninjured, typically developing comparison groups yielded similar findings.^32^ The only study to investigate children with mild TBI separately from moderate-to-severe TBI did not find any chronic (i.e. average of 2.8 years post-injury) differences between children with complicated mild TBI (i.e. classified based on the presence of neuroradiological findings) and those with extracranial injury.^29^ Combined, these results suggest that mild OI is a more conservative comparison than uninjured, typically developing children.

The current prospective, longitudinal study aimed to increase both scientific and clinical knowledge about the neurobiological effects of paediatric mild TBI by examining the structural connectome in children with mild TBI or mild OI. Specifically, we compared changes between the post-acute (2–33 days post-injury) and chronic (3 or 6 months post-injury) structural connectome of children with mild TBI or mild OI. Based on previous research,^29–33^ we hypothesized that, compared with children with mild OI, children with mild TBI would show less integrated and efficient network connectivity as indexed by reductions in the clustering coefficient (degree to which brain regions are connected to neighbouring regions), global efficiency (communication efficiency between distal network regions) and degree centrality (average connectivity among brain regions), together with increases in path length (the number of connections needed to travel between regions) and that these differences would show varying degrees of normalization across time (days) post-injury.

## Materials and methods

### Study design and procedure

Data were drawn from the Advancing Concussion Assessment in Paediatrics (A-CAP) study. Full details of this multisite study are provided elsewhere.^36^ The A-CAP study recruited children (8–16.99 years of age) within 48 h post-injury from five children’s hospital emergency departments in Canada, all members of the Pediatric Emergency Research Canada network.^36,37^ Children were assessed in the emergency department and also returned for a post-acute (i.e. targeted for 10 days post-injury; range 2–33 days) and two chronic (i.e. 3 and 6 months post-injury) assessments. Overall study attrition rates (i.e. 15, 25 and 28% for the post-acute, 3- and 6-month assessments, respectively) are consistent with other studies.^36^ Details about children’s acute clinical presentation were recorded in the emergency department. Mechanism of injury and a demographic questionnaire were collected at the post-acute follow-up.^36^ All participants in the study completed 3 T MRI at the post-acute follow-up and were randomly assigned to complete a second MRI scan at the 3 or 6 months (chronic) follow-up.

The study was conducted with the approval of the research ethics board at each study site and in accordance with the Declaration of Helsinki. All participants provided written informed assent and parents/guardians provided written informed consent.

### Participants

The participant inclusion and exclusion criteria has been previously described.^20,35,36,38,39^ Children with TBI sustained a blunt head trauma and met at least one of three criteria per the World Health Organization definition of mild TBI.^40^ Delayed neurological deterioration (e.g. Glasgow Coma Scale score <13), neurosurgical intervention, loss of consciousness >30 min or post-traumatic amnesia >24 h were exclusionary criteria for the TBI group.^36^ Children with OI sustained blunt force trauma to an upper or lower extremity that met criteria for mild OI based on the Abbreviated Injury Scale (i.e. score ≤4).^41^ Any children with suspected head trauma or signs of concussion were excluded from the OI group.^36^

### Symptoms

The Health and Behaviour Inventory, a core measure in the Common Data Elements for paediatric TBI,^42–44^ was used to assess cognitive and somatic symptoms. Parents rated premorbid (pre-injury) symptoms during the post-acute visit. Both parents and children rated post-injury symptoms weekly and also at each follow-up assessment.^45^ Premorbid and 1-month post-injury scores were compared to calculate a reliable change index (*z*-score) score. This approach is similar to that outlined in O’Brien *et al*.^44^ To develop the reliable change formulae, we regressed post-injury symptom scores for both parent and child rating onto parent premorbid (retrospective pre-injury) symptom scores separately for each post-injury assessment for the total scale scores. The resulting regression coefficients were then used to compute standardized change scores by subtracting predicted scores from actual post-injury scores and dividing by the standard error of the estimate. We then used the reliable change score results to classify children with mild TBI into two groups: (i) TBI with persistent symptoms (significant increase at 1-month post-injury relative to premorbid) and (ii) TBI without persistent symptoms (no significant increase at 1-month post-injury relative to premorbid).^46,47^

### Diffusion MRI

The whole-brain diffusion-weighted image acquisition protocol and approach to quality assurance^38^ was previously described.^36^ Whole-brain diffusion-weighted images were acquired using 30 different gradient directions at *b* = 900 s/mm^2^ and 5 at *b* = 0 s/mm^2^, with 2.2 mm isotropic resolution. Sites with General Electric scanners used repetition time (TR)/echo time (TE) = 6 s/70 ms (MR750; Montreal and Vancouver) or TR/TE = 12 s/90 ms (MR750w; Calgary); sites with Siemens scanners used TR/TE = 6.3/55 ms (Prisma; Edmonton and Montreal) or TR/TE = 7.8 s/90 ms (Skyra; Ottawa).

### Image processing

Diffusion-weighted image pre-processing procedures are detailed in our previous study,^39^ including the use of dcm2niix tool in MRIcron (https://github.com/rordenlab/dcm2niix) for DICOM to NIFTI conversion and the use of ExploreDTI v4.8.6 in MATLAB R2019a for correction of Gibb’s ringing, motion and eddy current artefacts.^48^

### Network construction

General network construction methods have been described previously.^35^ Briefly, ExploreDTI was used for deterministic whole-brain fibre tractography and to compute a connectivity matrix for each of the pre-processed diffusion-weighted images.^48^ A 90 × 90 node connectivity matrix was then computed for each scan. Nodes were defined in native space based on the Automated Anatomical Labeling 90 region atlas.^35,39,49^ Next, adjacency matrices were constructed using the average fractional anisotropy of fibre connections (passing fibres) among the 90 nodes.^48^ Connectivity matrices that were not fully connected were excluded (*n* = 20, 11 TBI/9 OI; see Supplementary Fig. 1).

### Network metrics

Weighted graph theoretical metrics for each scan and 1000 randomly generated networks were calculated in MATLAB R2019a using the GRaph thEoreTical Network Analysis toolbox v.2.0.^26^ Global and local (nodal) level metrics included degree, clustering coefficient, characteristic path length, small-worldness, betweenness and degree centrality, and efficiency (for definitions and descriptions, see Table 1).^26,50^

**Table 1:** Graph theory metrics for global and local network levels

Metric	Abbreviation (global/nodal)	Definition	Interpretation	Classification
Characteristic path length	Lp (λ)	Average shortest path length among all nodes within the network	Measure of integration efficiency between two nodes in the network	Integration
Efficiency	Eg/Ne	Strength of parallel information transfer among each node and all other nodes within the whole graph	Measure of integration, with higher efficiency indicating greater integration (via shorter path lengths) among a select brain region and other regions within the network	Integration
Clustering coefficient	Cp (γ)/NCp	Average proportion of node neighbours that are also considered to be neighbours	Overall extent to which proximal network regions are connected or clustered	Segregation
Betweenness centrality	Bc	Frequency that a node is part of average shortest paths		Centrality
Degree centrality	Dc	Number of edges (connections) of each node		Centrality
Small-worldness	Sigma (σ)	Ratio between the standardized clustering coefficient (γ) and shortest path length (λ)	Index of brain connectivity, or the presence of highly specialized and segregated regions, necessary for functional specialization, that are strongly connected to facilitate efficient integration and global network processing	

λ and γ are standardized against random networks. Definitions of global and local network parameters based on Rubinov and Sporns.^50^

### Scanner harmonization

Prior to final analysis, network metrics were harmonized for site (scanner) differences using neuroComBAT in RStudio v1.1.383 (R v4.0.3).^51–53^ Group, days (time) post-injury, age at injury and sex were included in the covariate matrix during harmonization. This approach can preserve the variability attributable to biological effects of interest (e.g. group, age, sex) while removing variability due to the use of different scanners.^39^

### Statistical analyses

Demographic data were analysed using *t*-tests for continuous variables and $\chi^{2}$ techniques for categorical variables.

Multiple linear mixed effects models were computed in RStudio using the lmerTest package to investigate the relations of group (TBI, OI), the linear and quadratic effects of time (days) post-injury, age at injury, sex and three-way interactions of group by time by age and group by time by sex on harmonized graph theory metrics, controlling for the random effect of participant.^52–55^ Hemisphere did not moderate group differences in preliminary analyses. Therefore, only the main effect of hemisphere was included in each model. The same approach was used to compare graph theory metrics between the children with TBI with persistent symptoms, TBI without persistent symptoms and OI (i.e. symptom status groups). The final model is given by the following formulas for global and nodal metrics, respectively:


(1) $$\mathrm{Global}\;\mathrm{metric}∼\mathrm{Group}\times (\mathrm{Time}+\mathrm{Tim}e^{2})\times (\mathrm{Age}+\mathrm{Sex})+(1|\mathrm{Participant})$$

or


(2) $$\mathrm{Regional}(\mathrm{nodal})\mathrm{metric}∼\mathrm{Group}\times (\mathrm{Time}+\mathrm{Tim}e^{2})\times (\mathrm{Age}+\mathrm{Sex})+\mathrm{Hemisphere}+(1|\mathrm{Participant})$$

Correction for multiple comparisons was conducted using the false discovery rate (FDR) at corrected *P* < 0.05 for group comparisons (i.e. TBI, OI) and corrected *P* < 0.025 for comparisons among symptom groups defined based on child and parent report (i.e. TBI with persistent symptoms, TBI without persistent symptoms, OI) to account for the use of both ratings.^56^ Standardized effect size (i.e. Cohen’s *d*) was assessed for group differences within the context of the final model for each network metric, with small, medium and large effect size indicated by |0.20| ≤ *d* < |0.50|, |0.50| ≤ *d* <|0.80| and *d* > |0.80|.^57^ Only effects with a 95% confidence interval range for *d* that excluded 0 were considered to be robust and are described below. Follow-up analyses for all significant interaction terms examined group differences at the average days post-injury at each assessment for interactions with time post-injury, in male and female children for interactions with sex and in younger (i.e. 10th percentile age at injury) and older (90th percentile age at injury) children for interactions with age at injury (see Supplementary Fig. 1).

The BrainNet Viewer toolbox was used in MATLAB R2019a to display nodal level results.^58^

## Results

### Sample

Information about the overall A-CAP study sample and the derivation of the current sample has been published^20^ and is summarized in Supplementary Fig. 1. Briefly, the final data set included 882 scans from 556 children after excluding 241 scans (21%; 170 TBI/71 OI) during initial quality assessment due to unstandardized acquisition parameters (104 TBI/40 OI), severe motion artefact (33 TBI/13 OI), incomplete acquisition (23 TBI/6 OI), scanner artefacts (5 TBI/4 OI), or gross brain structure abnormalities (2 TBI/4 OI). Twenty (11 TBI/9 OI) scans with partially connected connectivity matrices were excluded. The post-acute structural connectome of a small subset of the final sample (i.e. the Calgary cohort; 83 TBI/37 OI) was previously examined in relation to typical development.^35^

### Sociodemographic and injury characteristics

Mild TBI and OI groups differed in terms of injury mechanism, but not in other sociodemographic factors (e.g. age, sex, parental education, race or whether the injury was sustained during sport/recreation; see Table 2).

**Table 2:** Sample demographic and injury characteristics

Variable	Mild TBI	Mild OI	*P*
	*n* = 360	*n* = 196	
Study site [*n* (%)]			0.001
Calgary	103 (28.6)	45 (23.0)	
Edmonton	84 (23.3)	44 (22.4)	
Montreal	45 (12.5)	11 (5.6)	
Ottawa	43 (11.9)	19 (9.7)	
Vancouver	85 (23.6)	77 (39.3)	
Age [*Mean* (*SD*) years]	12.27 (2.44)	12.43 (2.23)	0.457
Sex [*n* (%) male]	223 (61.9)	109 (55.6)	0.173
Parental education [*n* (%)]			0.913
No certificate, diploma, or degree	11 (3.1)	4 (2.0)	
High school diploma or equivalent	51 (14.2)	24 (12.2)	
Trade certificate or diploma	35 (9.7)	16 (8.2)	
2-year college diploma	64 (17.8)	40 (20.4)	
4-year bachelor’s degree	125 (34.7)	63 (32.1)	
Master’s degree	37 (10.3)	24 (12.2)	
Doctoral degree (e.g. PhD or MD)	15 (4.2)	10 (5.1)	
Race [*n* (%)]			0.661
White	245 (68.1)	130 (66.3)	
Asian	30 (8.3)	13 (6.6)	
Black	15 (4.2)	6 (3.1)	
Latinx	8 (2.2)	8 (4.1)	
Indigenous	7 (1.9)	3 (1.5)	
Other/mixed	48 (13.3)	29 (14.8)	
Unknown	7 (1.9)	7 (3.6)	
Mechanism of injury [*n* (%)]			<0.001
Bicycle related	6 (1.7)	10 (5.1)	
Fall	136 (37.8)	90 (45.9)	
Motor vehicle collision	4 (1.1)	0 (0.0)	
Struck object	92 (25.6)	33 (16.8)	
Struck person	59 (16.4)	19 (9.7)	
Other	4 (1.1)	12 (6.1)	
Unknown	6 (1.7)	11 (5.6)	
Sport-related injury [*n* (%) sport/recreational play]	256 (71.1)	145 (74.0)	0.920
Loss of consciousness [*n* (%)]			
Yes	61 (16.9)		
Suspected	24 (6.7)		
Glasgow Coma Scale [*n* (%) = 15]^a^	326 (90.6)		
Prior concussion history [*n* (%)]^b^	57 (15.8)	34 (17.3)	0.147
Persistent symptoms 1-month post-injury [*n* (%)]			
Parent ratings	53 (14.7)		
Child ratings	66 (18.3)		

Uncorrected *P*-value reported. OI, orthopaedic injury; TBI, traumatic brain injury; SD, standard deviation. ^a^Glasgow Coma Scale scores 13 or greater. ^b^A total of 28 children (19 TBI/9 OI) had a prior history of concussion within 6 months of study enrolment.

### Network metrics

#### Global network

The injury groups did not differ in global network metrics.

#### Nodal network

**Figure 1:**
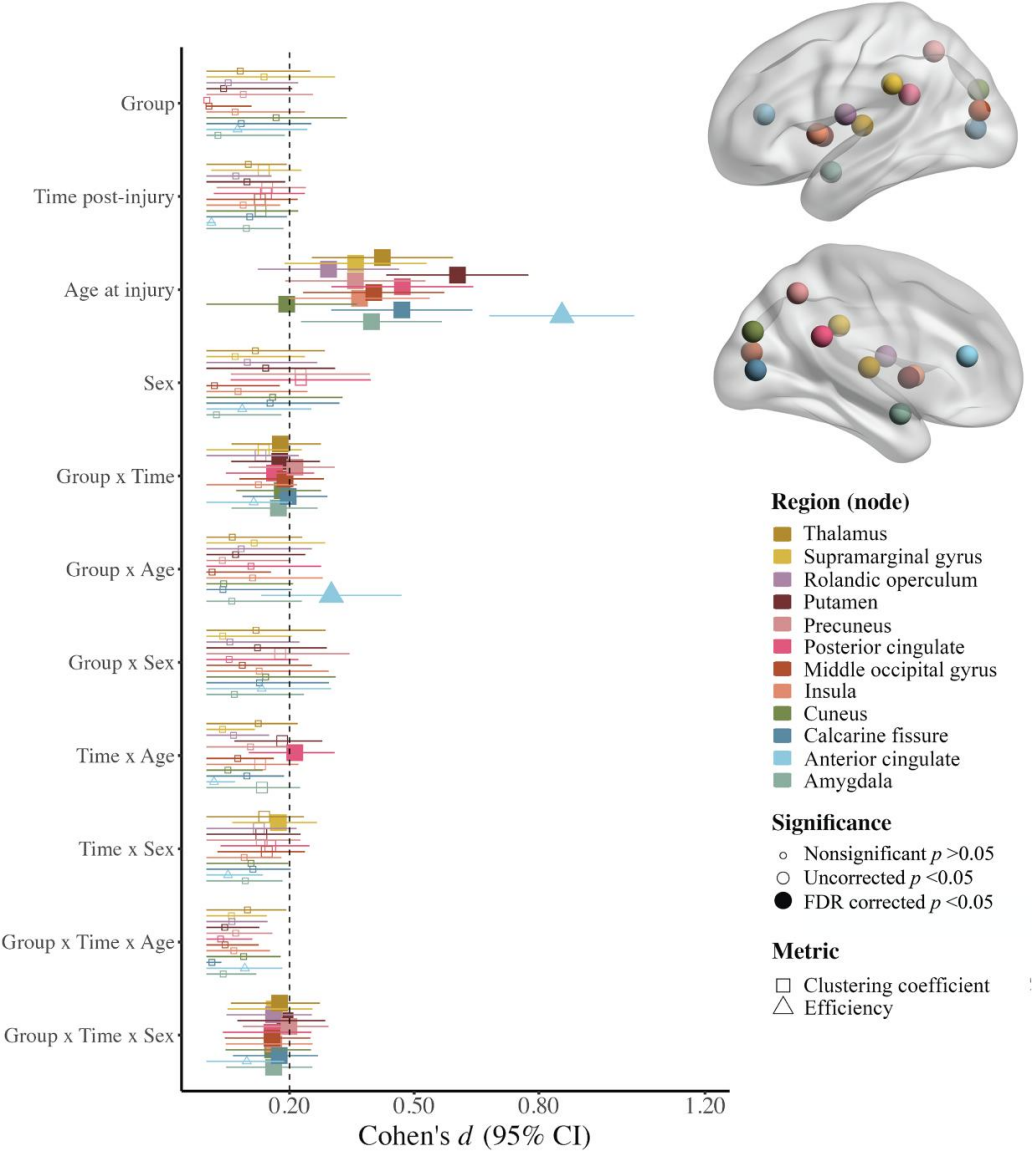
**Linear mixed effects models for regional (nodal) graph theory metrics with robust (i.e. FDR corrected *P* <0.05) injury group differences.** Standardized Cohen’s *d* (95% confidence interval) for model parameters of the linear mixed effects models used to examine regional graph theory metric differences between the (A) mild TBI (*n* = 360) and mild OI (*n* = 196) groups, with filled in shapes indicative of significance after multiple comparisons (see legend; *FDR corrected *P* < 0.05, shape reflects the graph theory metric and colour reflects the region of interest where effects were noted (see legend). Regions where neither main nor interaction effects of group were significant (i.e. FDR corrected *P* > 0.05) are not shown here. All models included covariate hemisphere.

**Figure 2:**
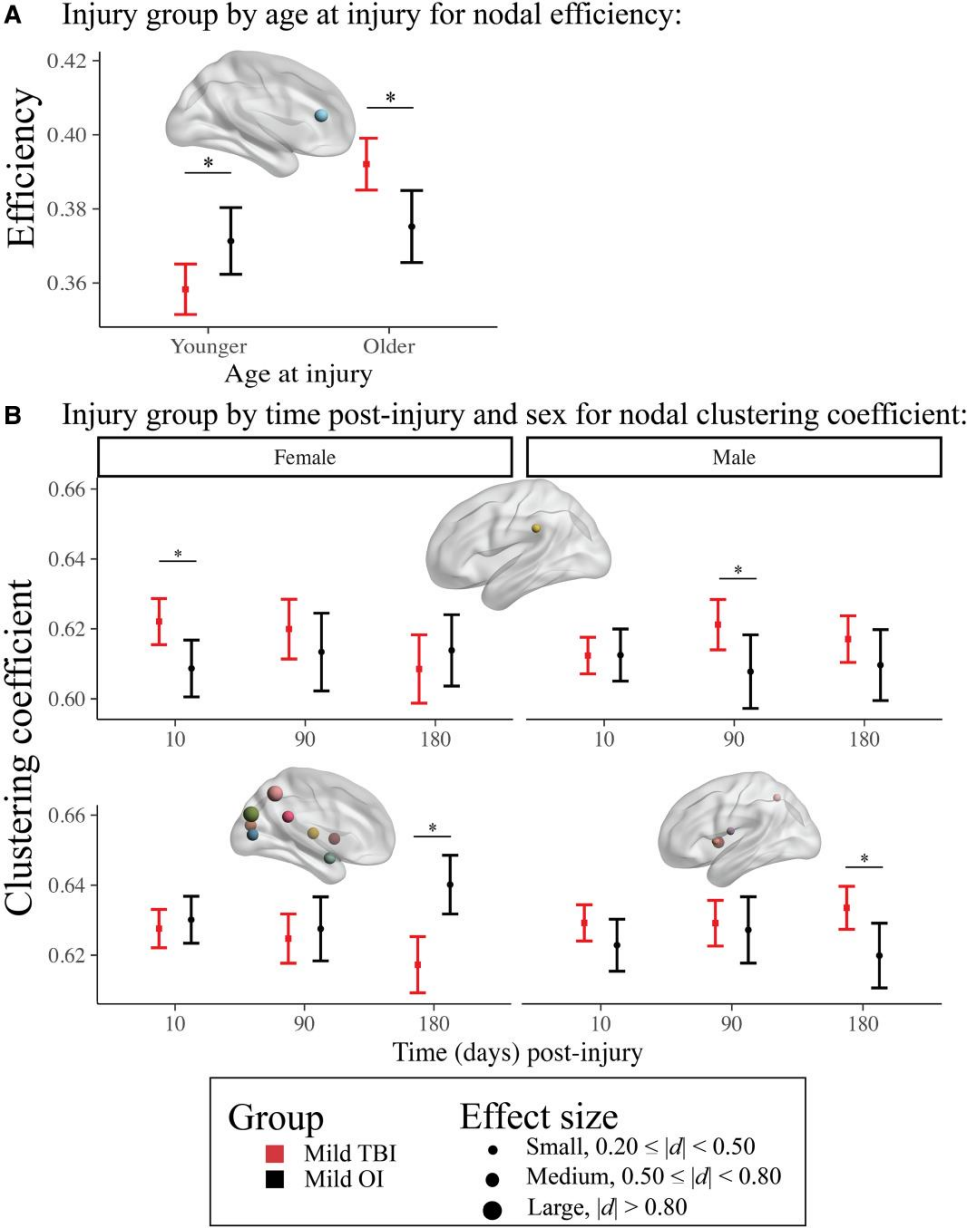
**Differences in regional (nodal) network metrics between injury groups were moderated by age at injury and by time post-injury and sex.** Graphs illustrate the moderating effects of (**A**) age at injury for anterior cingulate efficiency and (**B**) time post-injury and biological sex for differences in regional clustering coefficient of several regions between the children with mild (*n* = 360) and mild OI (*n* = 196). For (**B**), the top graph shows post-acute injury group differences in females (left) and 3 months group differences in males (right); the bottom graph shows group differences in clustering coefficient 6 months post-injury for females (left) and males (right). Robust differences between the injury groups (i.e. Cohen’s *d* 95% confidence interval excluded 0) are denoted by *. For each effect, brain regions that showed the illustrated pattern between the injury groups are shown in the brain image insets for each graph (see Fig. 1 legends). For robust effects (i.e. *d* 95% confidence interval excluded 0), Cohen’s *d* is denoted by node size. Younger corresponds to the 10th percentile age at injury; Older corresponds to 90th percentile age at injury.

Regional (nodal) network measures with injury group effects that survived correction for multiple comparisons are illustrated in Fig. 1 (for statistical results see Supplementary Table 1). Age moderated injury group differences in efficiency of the anterior cingulate. Specifically, efficiency was lower after TBI relative to OI in younger children, while the opposite pattern (i.e. higher efficiency) was observed after TBI in older children, across time post-injury (Fig. 2A). Time and sex moderated group differences in the clustering coefficient for the posterior cingulate, supramarginal gyrus, cuneus, precuneus, Rolandic operculum, insula, middle occipital gyrus, calcarine fissure, amygdala, putamen and thalamus (Fig. 2B). Specifically, the clustering coefficient of the supramarginal gyrus was higher after TBI relative to OI post-acutely in females and at 3 months post-injury in males. However, it was lower for the putamen, thalamus, amygdala, cuneus, posterior cingulate, precuneus, middle occipital and calcarine fissure 6 months after TBI relative to OI in females, but higher for the putamen, Rolandic operculum, insula and precuneus 6 months after TBI in males.

#### Persistent symptoms

Global metrics did not differ among persistent symptom groups based on child or parent report. However, differences were observed in regional (nodal) metrics among symptom groups based on parent and child report that were moderated by time, sex and age (see below).

#### Child reported symptoms

**Figure 3:**
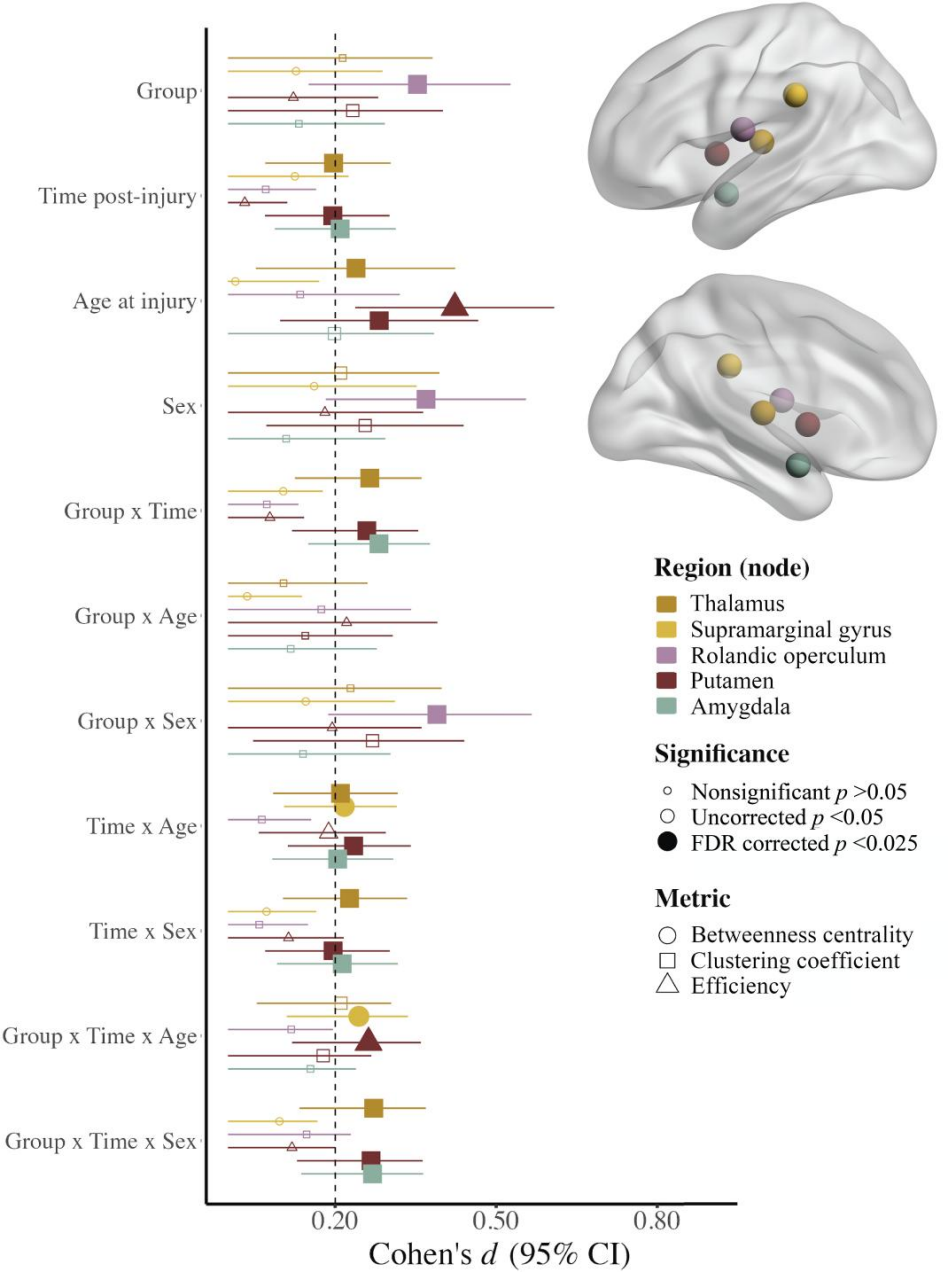
**Linear mixed effects models for regional (nodal) graph theory metrics with robust (i.e. FDR corrected *P* <0.025) differences between symptom groups based on child report.** Standardized Cohen’s *d* (95% confidence interval) for model parameters of the linear mixed effects models used to examine regional graph theory metric differences between the symptom groups based on child report. Size reflects the significance of the effect, with filled in shapes indicative of significance after multiple comparisons (see legend; *FDR corrected *P* <0.025), shape reflects the graph theory metric and colour reflects the region of interest where effects were noted (see legend). Regions where neither main nor interaction effects of group were significant (i.e. FDR corrected *P* >0.025) are not shown here. All models included covariate hemisphere.

**Figure 4:**
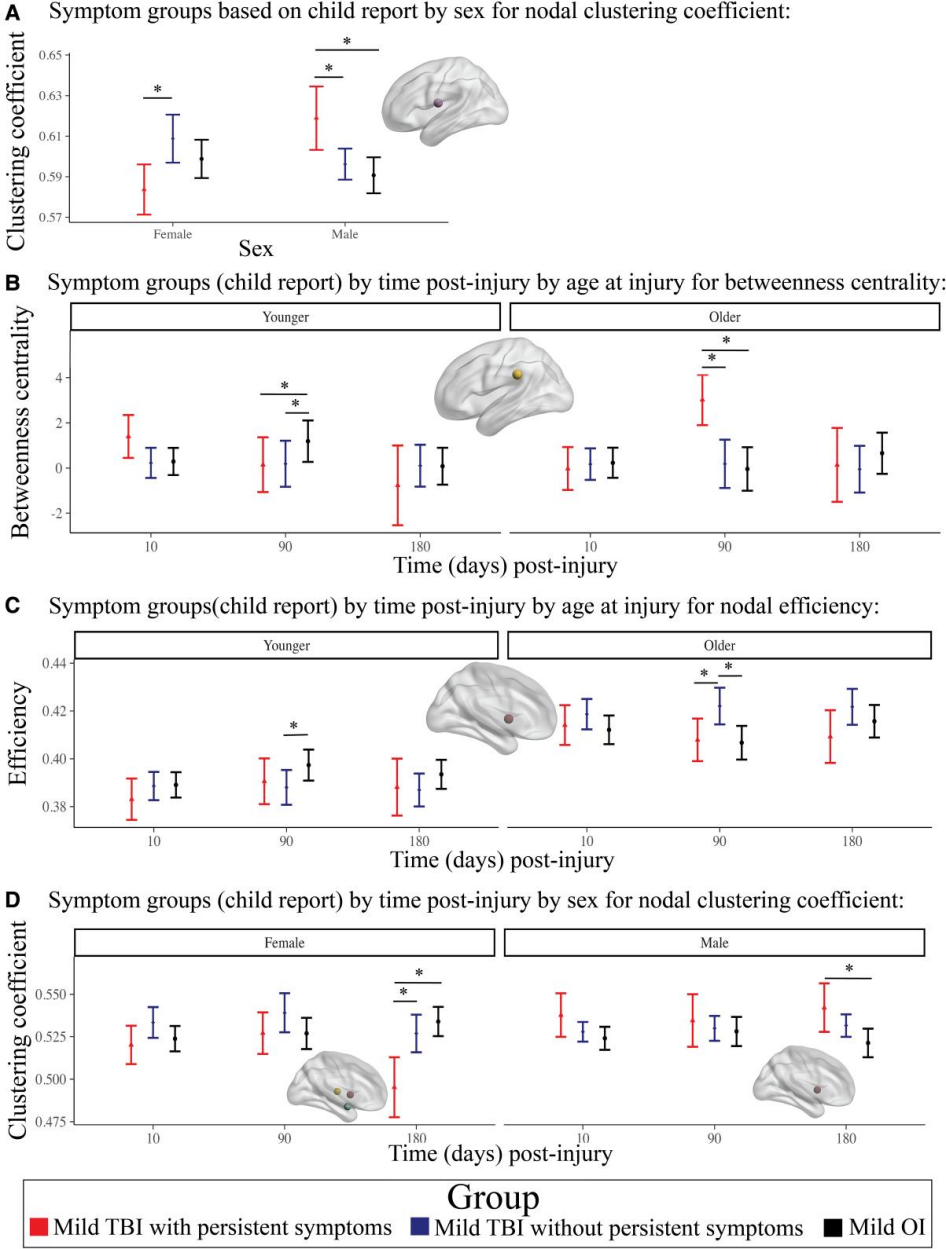
**Differences in regional (nodal) graph metrics among symptom groups based on child report were moderated by time post-injury, age at injury and biological sex.** Graphs illustrate the moderating effects of (**A**) sex for Rolandic operculum clustering coefficient, (**B**) time post-injury and age at injury for differences in supramarginal gyrus betweenness centrality, (**C**) time post-injury and age at injury for nodal efficiency of several regions and (**D**) time post-injury and sex for nodal clustering coefficient of several regions between the children with mild TBI with persistent symptoms (*n* = 66), mild TBI without persistent symptoms (*n* = 205) and OI (*n* = 196). For **B** and **C**, group differences are shown for younger children (left) and older children (right). For **D**, group differences are shown for females (left) and males (right). Graphs illustrate robust differences between the groups (i.e. Cohen’s *d* 95% confidence interval excluded 0), as denoted by *. For each effect, brain regions that showed the illustrated pattern between the groups are shown in the brain image insets for each graph (see Fig. 3 legends).

The final model results for symptom groups based child report with group effects that survived FDR correction are illustrated in Fig. 3 (for statistical results, see Supplementary Table 2). Across time post-injury, the clustering coefficient of the Rolandic operculum was lower in females with TBI with persistent symptoms (*n* = 66) relative to those with TBI without persistent symptoms (*n* = 205), but was higher in males with TBI with persistent symptoms relative to TBI without persistent symptoms and OI (Fig. 4A). Regional metric differences among symptom groups based on child report were moderated by time and sex or age (Fig. 4B–D). Specifically, at 3 months post-injury, betweenness centrality of the supramarginal gyrus was lower in TBI with and without persistent symptoms relative to OI in younger children, but was higher in TBI with persistent symptoms relative to TBI without persistent symptoms and OI in older children. Efficiency of the putamen was lower in TBI without persistent symptoms relative to OI in younger children, but was higher in TBI without persistent symptoms relative to TBI with persistent symptoms and OI in older children. At 6 months post-injury, clustering coefficient of the amygdala, thalamus and putamen was lower in TBI with persistent symptoms relative to TBI without persistent symptoms and OI in females, whereas clustering coefficient of the putamen was higher in TBI with persistent symptoms relative to OI in males.

#### Parent reported symptoms

**Figure 5:**
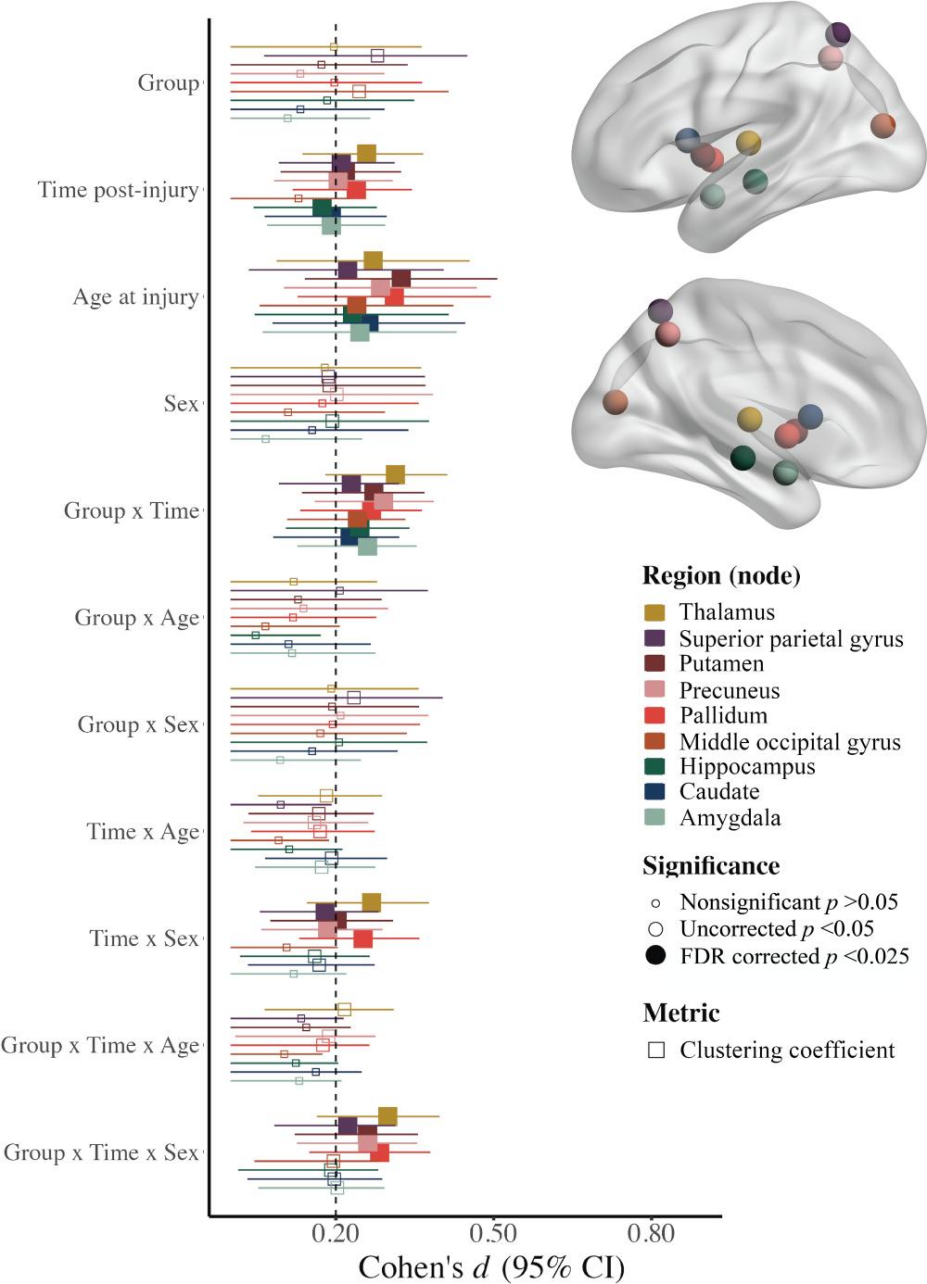
**Linear mixed effects models for regional (nodal) graph theory metrics with robust (i.e. FDR corrected *P* <0.025) differences between symptoms groups based on parent report.** Standardized Cohen’s *d* (95% confidence interval) for model parameters of the linear mixed effects models used to examine regional graph theory metric differences between the symptom groups based on parent report. Size reflects the significance of the effect, with filled in shapes indicative of significance after multiple comparisons (see legend; *FDR corrected *P* <0.025), shape reflects the graph theory metric, and colour reflects the region of interest where effects were noted (see legend). Regions where neither main nor interaction effects of group were significant (i.e. FDR corrected *P* >0.025) are not shown here. All models included covariate hemisphere.

**Figure 6:**
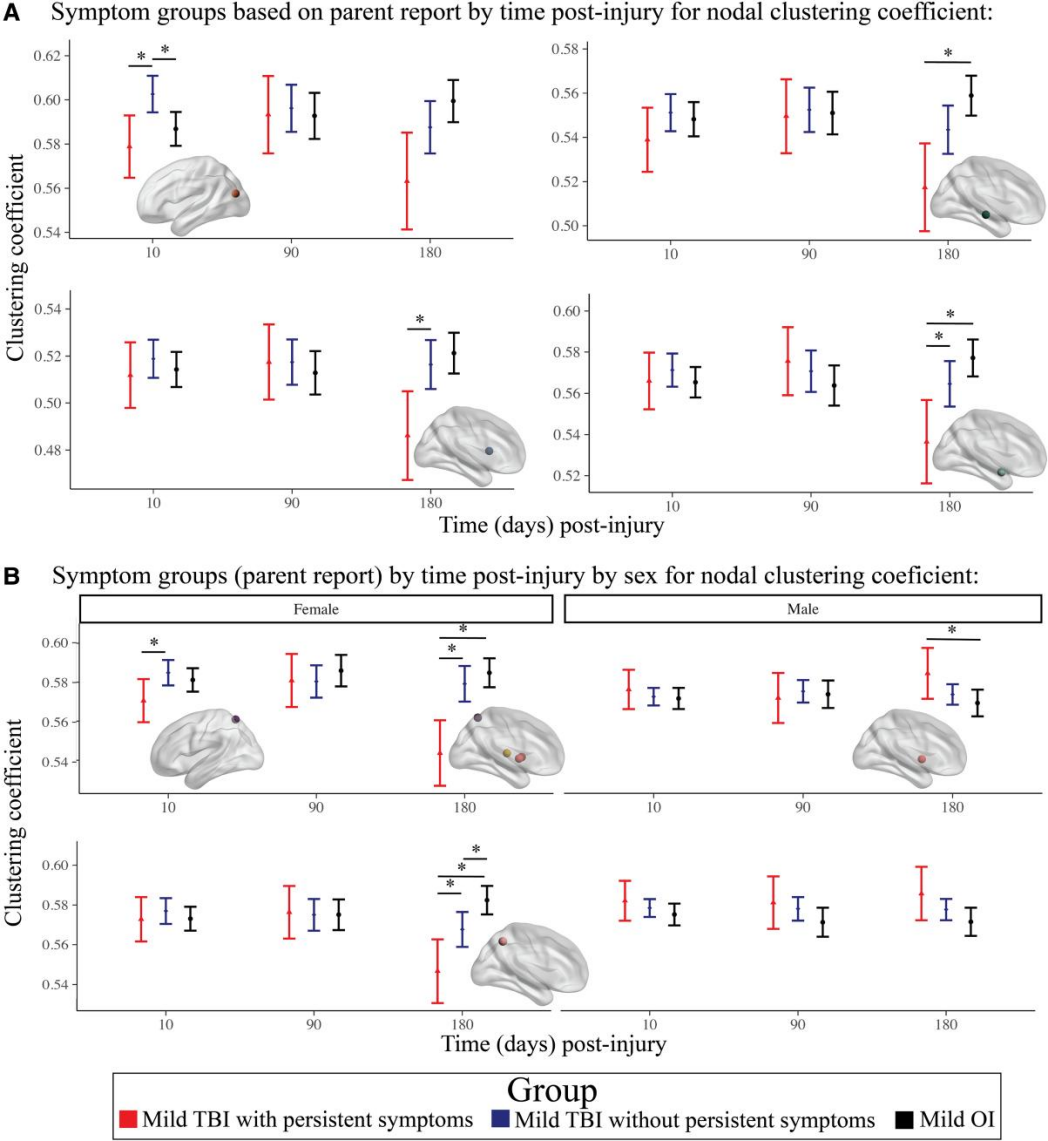
**Differences in regional (nodal) graph metrics among symptom groups based on parent report were moderated by time post-injury and biological sex.** Graphs illustrate the moderating effects of (**A**) time post-injury for nodal clustering coefficient of several regions and (**B**) time post-injury and biological sex for differences in nodal clustering coefficient of several regions between the children with mild TBI with persistent symptoms (*n* = 53), mild TBI without persistent symptoms (*n* = 230) and OI (*n* = 196). For **B**, group differences that were demonstrated post-acutely and at 6 months post-injury are shown for females (top left) and males (top right); the bottom right shows results for group differences that were demonstrated at 6 months post-injury only in females (bottom right). Graphs illustrate robust differences between the groups (i.e. Cohen’s *d* 95% confidence interval excluded 0), as denoted by *. For each effect, brain regions that showed the illustrated pattern between the groups are shown in the brain image insets for each graph (see Fig. 5 legends).

The final model results for symptom groups based parent report with group effects that survived FDR correction are illustrated in Fig. 5 (for statistical results, see Supplementary Table 2). Regional metrics differed among symptom groups based on parent report over time (Fig. 6A). Specifically, the clustering coefficient of the middle occipital gyrus was higher in TBI without persistent symptoms (*n* = 230) relative to TBI with persistent symptoms (*n* = 53) and OI post-acutely. At 6 months post-injury, clustering coefficient was lower in TBI with persistent symptoms relative to TBI without persistent symptoms for the caudate and amygdala, and relative to OI for the hippocampus and amygdala.

Regional metric differences also differed by time and sex (Fig. 6B). The clustering coefficient of the superior parietal gyrus was lower post-acutely in females with TBI with persistent symptoms relative to TBI without persistent symptoms. At 6 months post-injury, clustering coefficient was lower for several regions (i.e. thalamus, pallidum, putamen, precuneus, superior parietal gyrus) in females with TBI with persistent symptoms relative to TBI without persistent symptoms and OI, and for the precuneus in females with TBI without persistent symptoms relative to OI. In contrast, the clustering coefficient of the pallidum was greater in males with TBI with persistent symptoms relative to OI 6 months post-injury.

## Discussion

In the largest study of the structural connectome in children with mild TBI to date, we showed that regional (nodal) and local structural network topology alterations were apparent following mild TBI and were moderated by symptom persistence at 1-month post-injury, time post-injury, age at injury and biological sex. Alterations of regional network parameters were more robust and widespread at chronic timepoints as opposed to post-acutely after mild TBI. These regional differences and the absence of global brain network differences, suggest subtle alterations following mild TBI that are apparent up to 6 months post-injury and vary by sex and age at injury. These results build on prior tractography studies of white-matter microstructure reported in overlapping (i.e. the A-CAP study)^20,35^ and independent samples, which also show more chronic than early alterations.^17–19^

Post-acute regional network metrics distinguished mild TBI from mild OI and were associated with symptom recovery 1-month post-injury. While prognostic, the small to modest effect size of post-acute group differences and the moderating effects of sex likely would preclude effective clinical application in individual patients. Moreover, the predictive utility of network metrics did not extend to global metrics, but was isolated to regions of the parietal and occipital cortex. Lower clustering coefficient post-acutely after mild TBI in children who showed persistent symptoms at 1-month post-injury suggests that altered segregation of specialized, local networks and connectivity among neural subnetworks can occur early and relate to persistent symptoms following mild TBI, albeit differently for males and females.

Longitudinal changes in regional and local subnetwork structure were moderated by sex and age at injury. In typical development, network changes (e.g. increasing global efficiency and decreasing local efficiency, clustering coefficient and characteristic path length) likely reflect strengthening white-matter connectivity and grey matter pruning that occur into young adulthood and allow for increasingly efficient information processing and integration.^50,59–61^ Graph theory metric differences also exist between typically developing males and females.^62^ Here, females with mild TBI (both with and without persistent symptoms) demonstrated reduced clustering coefficient in several cortical and subcortical grey matter structures at chronic periods post-injury; and older children with TBI and persistent symptoms had higher betweenness centrality and reduced nodal efficiency. This pattern suggests that increased segregation (modularity) and reduced efficiency of local networks emerges over time and persists up to 6 months after mild TBI, especially in females and adolescents with persistent symptoms.

The emergence of changes in specific metrics (nodal efficiency, characteristic path length, betweenness centrality) and additional regions at chronic periods post-injury provides further evidence that mild TBI causes changes in connectivity that are both region and feature (metric) dependent.^31,34^ Specifically, our results contribute to mounting evidence that structural reorganization, in this case, segregated and inefficient network topology, of white-matter connectivity among parietal, limbic, occipital and subcortical grey matter regions may characterize mild TBI and contibute to prolonged recovery in children.^32,63^ Existing neuroimaging studies have consistently demonstrated structural and functional alterations in cortical and subcortical brain regions similar to those demonstrated here, including in the parietal lobe,^17,64–66^ occipital lobe,^67^ thalamus, basal ganglia, amygdala and hippocampus.^17^ We recently reported that children in the A-CAP study with mild TBI (across symptom groups and in children with persistent symptoms) showed chronic post-injury white-matter microstructural alterations (i.e. higher mean diffusivity) of the superior longitudinal fasciculus (i.e. a major association tract that connects parietal and frontal cortices) and anterior thalamic radiations (i.e. a fibre tract that connects the prefrontal cortex, limbic system, striatum and thalamus).^20^

The lack of group differences in global network structure across time post-injury was unexpected. We had initially predicted that the diffuse, microscopic alterations characteristic of mild TBI would disrupt connectivity both within and between regions of specialized brain subnetworks, culminating in the largely non-focal symptoms of mild TBI. However, our overall results suggest that mild TBI causes more focal and minor local network changes instead of altering global network structure. A study in adults with mild TBI similarly found regional and local network disruption with no evidence of global network alterations.^68^ Segregated and inefficient network topology has previously been demonstrated in early and late periods after paediatric mild TBI, at both the global and local structural network levels.^29–33^ Global network metrics differ in the first few weeks after mild traumatic injury (i.e. both mild TBI and mild OI) compared with typical development, although the specific global metrics reported across studies have varied and are not always altered specifically after paediatric mild TBI.^29^ Methodological inconsistencies and limitations of past research could explain discrepancies between our results and studies that found altered global metrics in the post-acute period. For instance, children with OI provide a more conservative comparison group than the uninjured, typically developing children (i.e. ‘controls’) examined in other studies and could help account for the lack of global differences demonstrated between groups and the generally subtle local differences observed currently.^29,32,35^

The overall pattern of results for symptom groups differed when based on child when compared with parent reports. This is not surprising when considering generally modest agreement between child and parent ratings.^44,69^ Specifically, O’Brien *et al*.^44^ found that child and parent mean ratings differed for somatic symptoms but did not differ for cognitive symptoms, across time post-injury. While the regions and timing of network metric differences between symptom groups overlapped somewhat, the findings based on parent report included a greater number of cortical brain regions that also were more distributed throughout the brain relative to the results for symptom groups based on child report (see Figs 3 and 4).

### Limitations

The A-CAP sample was recruited from the emergency departments of paediatric hospitals and may not be representative of patients who do not seek acute medical care or who are seen in other settings, such as primary care. The sample also may not generalize to children with lower socioeconomic status. Subconcussive brain injury cannot be entirely excluded in the children with OI,^70–72^ although children in the OI group with any head trauma or signs or symptoms of concussion were excluded from the study. This is an important consideration for future research given that most injuries in this sample occurred in sport or recreational settings. Weighted fractional anisotropy values were examined, but other matrix construction methods (e.g. binarized; streamline count) or the use of multi-shell diffusion-weighted MRI could yield different conclusions about group differences. Finally, we compared MRI metrics among groups based on symptom persistence at 1-month post-injury relative to premorbid symptoms in the children with mild TBI.^46^ Further examination of more specific symptom profiles (e.g. cognitive versus somatic symptoms) over time in relation to structural brain networks would further our understanding of the neurobiological outcomes of paediatric mild TBI, and may be especially important given initial evidence that structural connectivity can be influenced by intervention and may moderate post-injury symptom trajectory.^73^

## Conclusions

This multisite prospective, longitudinal dual cohort study examined trajectories of change in structural brain networks after paediatric mild TBI as compared with mild OI in the largest sample to date. Local network and regional (nodal) alterations in structural connectivity were apparent within the first few weeks post-injury and were followed by dynamic trajectories of changes up to 6 months following paediatric mild TBI. Group differences were influenced by symptom persistence at 1-month post-injury, time post-injury, biological sex and age at injury.^17,64^ These findings highlight the importance of contextualizing paediatric mild TBI and its outcomes within a neurodevelopmental framework. While prognostic, the small to modest effect size of group differences and the moderating effects of sex likely would preclude effective clinical application in individual patients.

## Supplementary Material

fcad173_Supplementary_Data

## Data Availability

A data set with deidentified participant data and a data dictionary will be made available upon reasonable request from any qualified investigator, subject to a signed data access agreement.

## Abbreviations

A-CAP: Advancing Concussion Assessment in Paediatrics
FDR: false discovery rate
OI: orthopaedic injury
TBI: traumatic brain injury
TE: echo time
TR: repetition time.
