# Supplementary material for: Altered longitudinal structural connectome in paediatric mild traumatic brain injury: an Advancing Concussion Assessment in Paediatrics study
Source: Brain Commun. 2023 May 31;5(3):fcad173. doi: 10.1093/braincomms/fcad173 (PMC10265725; doi:10.1093/braincomms/fcad173)
Supplement: fcad173_Supplementary_Data [file fcad173_supplementary_data.docx]

***Supplementary Figure 1* Summary data for the overall A-CAP study sample and the derivation of the current sample.** Of 3,075 eligible children with mild traumatic brain injury (TBI) or mild orthopedic injury (OI), 967 consented to participate in the A-CAP study, and 846 returned for at least one assessment. Children who returned at post-acute, 3 months, or 6 months did not differ from those who did not return in terms of age, sex, race, or parental education, with one exception: children who returned at 6 months had higher parental education than those who did not return. Overall, 671 children completed at least one MRI as part of the A-CAP study, with a total of 1144 scans completed. However, 241 scans (21%; 170 TBI/71 OI) were excluded during initial quality assessment due to: unstandardized acquisition parameters (104 TBI/40 OI), severe motion artifact (33 TBI/13 OI), incomplete acquisition (23 TBI/6 orthopedic injury), scanner artifacts (5 TBI/4 OI), or gross brain structure abnormalities (2 TBI/4 OI). Data from 20 (11 TBI/9 OI) scans had partially connected connectivity matrices and were also excluded, resulting in a final dataset of 882 diffusion-weighted MRI scans from 556 children (see Table 2).

*
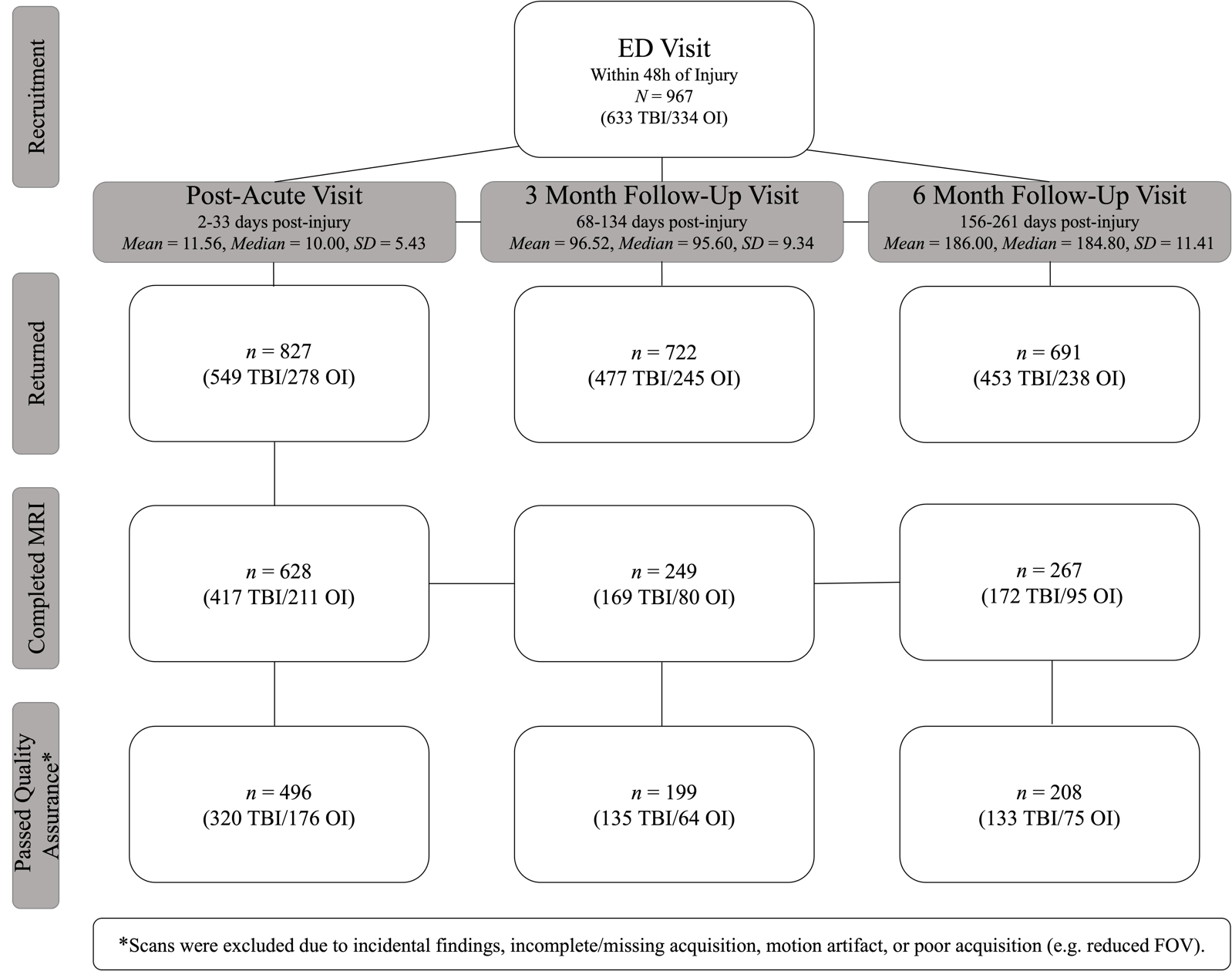
*

*Note.* OI = orthopedic injury; TBI = traumatic brain injury.

***Supplemental Table 1*** **Follow-up pairwise comparisons for significant differences (FDR corrected *P* < .05) in DTI metrics between injury groups (see Fig. 1-2).** Pairwise comparisons conducted within the context of the final fitted model.

|  |  |  |  |  | ***Estimated Marginal Mean (95% CI)*** | | **TBI – OI** | | |  |
| --- | --- | --- | --- | --- | --- | --- | --- | --- | --- | --- |
| ***Metric*** | ***Region (node)*** | ***Time (days) post-injury*** | ***Age at injury*** | ***Sex*** | ***Mild TBI*** | ***Mild OI*** | ***Cohen’s d*** | ***df*** | ***SE*** |  |
| Efficiency (Ne) | | | | | | | | | | |
|  | Anterior cingulum | - | Younger  (10^th^ percentile) | - | 0.35 (0.35, 0.36) | 0.36 (0.35, 0.37) | -0.61 (-1.13, -0.09)* | 1224.57 | 0.27 |  |
|  |  | - | Older  (90^th^ percentile) | - | 0.39 (0.38, 0.39) | 0.37 (0.36, 0.37) | 1.02 (0.49, 1.55)* | 1091.13 | 0.27 |  |
| Clustering coefficient (NCp) | | | | | | | | | | |
|  | Posterior cingulum | 10 | - | Female | 0.63 (0.62, 0.64) | 0.63 (0.62, 0.64) | 0.12 (-0.28, 0.52) | 714.90 | 0.20 |  |
|  |  |  | - | Male | 0.64 (0.63, 0.64) | 0.64 (0.63, 0.64) | 0.03 (-0.31, 0.38) | 724.99 | 0.17 |  |
|  |  | 90 | - | Female | 0.63 (0.63, 0.64) | 0.63 (0.61, 0.64) | 0.31 (-0.20, 0.81) | 1224.89 | 0.26 |  |
|  |  |  | - | Male | 0.64 (0.63, 0.65) | 0.63 (0.62, 0.64) | 0.32 (-0.14, 0.77) | 1324.68 | 0.23 |  |
|  |  | 180 | - | Female | 0.62 (0.61, 0.63) | 0.63 (0.62, 0.64) | -0.55 (-1.06, -0.04)* | 1220.32 | 0.26 |  |
|  |  |  | - | Male | 0.64 (0.64, 0.65) | 0.63 (0.62, 0.64) | 0.30 (-0.14, 0.73) | 1226.46 | 0.22 |  |
|  | Supramarginal gyrus | 10 | - | Female | 0.61 (0.61, 0.62) | 0.60 (0.59, 0.61) | 0.42 (0.10, 0.74)* | 814.75 | 0.16 |  |
|  |  |  | - | Male | 0.60 (0.60, 0.61) | 0.60 (0.60, 0.61) | 0.00 (-0.28, 0.27) | 822.82 | 0.14 |  |
|  |  | 90 | - | Female | 0.61 (0.60, 0.62) | 0.60 (0.59, 0.61) | 0.20 (-0.23, 0.64) | 1327.16 | 0.22 |  |
|  |  |  | - | Male | 0.61 (0.60, 0.62) | 0.60 (0.59, 0.61) | 0.42 (0.03, 0.81)* | 1405.70 | 0.20 |  |
|  |  | 180 | - | Female | 0.60 (0.59, 0.61) | 0.60 (0.59, 0.61) | -0.17 (-0.60, 0.27) | 1310.97 | 0.22 |  |
|  |  |  | - | Male | 0.61 (0.60, 0.61) | 0.60 (0.59, 0.61) | 0.23 (-0.14, 0.60) | 1319.43 | 0.19 |  |
|  | Rolandic operculum | 10 | - | Female | 0.60 (0.60, 0.61) | 0.59 (0.59, 0.60) | 0.28 (-0.05, 0.61) | 801.98 | 0.17 |  |
|  |  |  | - | Male | 0.60 (0.60, 0.61) | 0.60 (0.59, 0.61) | 0.08 (-0.20, 0.36) | 810.57 | 0.14 |  |
|  |  | 90 | - | Female | 0.60 (0.59, 0.61) | 0.61 (0.60, 0.62) | -0.21 (-0.65, 0.23) | 1320.44 | 0.22 |  |
|  |  |  | - | Male | 0.61 (0.60, 0.61) | 0.60 (0.59, 0.61) | 0.36 (-0.04, 0.76) | 1401.95 | 0.20 |  |
|  |  | 180 | - | Female | 0.59 (0.59, 0.60) | 0.60 (0.59, 0.61) | -0.07 (-0.52, 0.37) | 1305.12 | 0.23 |  |
|  |  |  | - | Male | 0.61 (0.60, 0.61) | 0.59 (0.59, 0.60) | 0.41 (0.04, 0.79)* | 1313.31 | 0.19 |  |
|  | Insula | 10 | - | Female | 0.55 (0.54, 0.56) | 0.55 (0.54, 0.56) | 0.04 (-0.34, 0.42) | 737.17 | 0.19 |  |
|  |  |  | - | Male | 0.55 (0.55, 0.56) | 0.55 (0.54, 0.56) | 0.07 (-0.25, 0.40) | 747.26 | 0.16 |  |
|  |  | 90 | - | Female | 0.55 (0.54, 0.56) | 0.56 (0.55, 0.57) | -0.21 (-0.69, 0.28) | 1259.70 | 0.25 |  |
|  |  |  | - | Male | 0.55 (0.55, 0.56) | 0.55 (0.54, 0.56) | 0.25 (-0.19, 0.69) | 1355.59 | 0.22 |  |
|  |  | 180 | - | Female | 0.54 (0.53, 0.55) | 0.56 (0.55, 0.56) | -0.48 (-0.97, 0.00) | 1251.61 | 0.25 |  |
|  |  |  | - | Male | 0.55 (0.55, 0.56) | 0.54 (0.53, 0.55) | 0.47 (0.05, 0.89)* | 1258.34 | 0.21 |  |
|  | Cuneus | 10 | - | Female | 0.62 (0.61, 0.63) | 0.63 (0.62, 0.63) | -0.10 (-0.42, 0.23) | 805.70 | 0.17 |  |
|  |  |  | - | Male | 0.63 (0.62, 0.63) | 0.62 (0.62, 0.63) | 0.05 (-0.23, 0.33) | 814.15 | 0.14 |  |
|  |  | 90 | - | Female | 0.62 (0.61, 0.63) | 0.62 (0.61, 0.63) | -0.11 (-0.55, 0.33) | 1322.54 | 0.22 |  |
|  |  |  | - | Male | 0.63 (0.62, 0.63) | 0.63 (0.62, 0.64) | -0.10 (-0.49, 0.30) | 1403.18 | 0.20 |  |
|  |  | 180 | - | Female | 0.61 (0.60, 0.62) | 0.64 (0.63, 0.65) | -0.88 (-1.32, -0.44)* | 1306.95 | 0.22 |  |
|  |  |  | - | Male | 0.63 (0.62, 0.63) | 0.62 (0.61, 0.63) | 0.14 (-0.24, 0.51) | 1315.22 | 0.19 |  |
|  | Precuneus | 10 | - | Female | 0.56 (0.55, 0.56) | 0.55 (0.55, 0.56) | 0.07 (-0.32, 0.46) | 723.35 | 0.20 |  |
|  |  |  | - | Male | 0.56 (0.56, 0.57) | 0.56 (0.55, 0.56) | 0.22 (-0.11, 0.56) | 733.47 | 0.17 |  |
|  |  | 90 | - | Female | 0.55 (0.55, 0.56) | 0.56 (0.55, 0.57) | -0.10 (-0.59, 0.40) | 1239.16 | 0.25 |  |
|  |  |  | - | Male | 0.56 (0.56, 0.57) | 0.55 (0.54, 0.56) | 0.41 (-0.04, 0.86) | 1337.59 | 0.23 |  |
|  |  | 180 | - | Female | 0.54 (0.54, 0.55) | 0.57 (0.56, 0.58) | -0.87 (-1.37, -0.37)* | 1233.19 | 0.26 |  |
|  |  |  | - | Male | 0.56 (0.56, 0.57) | 0.55 (0.54, 0.56) | 0.44 (0.02, 0.87)* | 1239.57 | 0.22 |  |
|  | Middle occipital gyrus | 10 | - | Female | 0.59 (0.58, 0.60) | 0.58 (0.57, 0.59) | 0.26 (-0.07, 0.58) | 807.40 | 0.17 |  |
|  |  |  | - | Male | 0.58 (0.58, 0.59) | 0.58 (0.57, 0.59) | 0.16 (-0.12, 0.44) | 815.78 | 0.14 |  |
|  |  | 90 | - | Female | 0.59 (0.58, 0.60) | 0.59 (0.58, 0.60) | 0.01 (-0.43, 0.45) | 1323.46 | 0.22 |  |
|  |  |  | - | Male | 0.59 (0.58, 0.59) | 0.58 (0.57, 0.59) | 0.29 (-0.10, 0.69) | 1403.71 | 0.20 |  |
|  |  | 180 | - | Female | 0.58 (0.57, 0.59) | 0.59 (0.58, 0.60) | -0.55 (-0.99, -0.11)* | 1307.75 | 0.22 |  |
|  |  |  | - | Male | 0.59 (0.58, 0.59) | 0.58 (0.57, 0.59) | 0.24 (-0.14, 0.62) | 1316.06 | 0.19 |  |
|  | Calcarine fissure | 10 | - | Female | 0.59 (0.58, 0.60) | 0.59 (0.58, 0.59) | 0.12 (-0.23, 0.47) | 772.23 | 0.18 |  |
|  |  |  | - | Male | 0.59 (0.59, 0.60) | 0.59 (0.58, 0.60) | 0.11 (-0.19, 0.41) | 781.76 | 0.15 |  |
|  |  | 90 | - | Female | 0.59 (0.58, 0.60) | 0.59 (0.58, 0.61) | -0.22 (-0.68, 0.24) | 1298.81 | 0.23 |  |
|  |  |  | - | Male | 0.59 (0.59, 0.60) | 0.59 (0.58, 0.60) | 0.14 (-0.27, 0.56) | 1387.17 | 0.21 |  |
|  |  | 180 | - | Female | 0.58 (0.57, 0.59) | 0.60 (0.59, 0.61) | -0.72 (-1.18, -0.25)* | 1286.22 | 0.24 |  |
|  |  |  | - | Male | 0.60 (0.59, 0.60) | 0.59 (0.58, 0.60) | 0.29 (-0.11, 0.68) | 1293.78 | 0.20 |  |
|  | Putamen | 10 | - | Female | 0.52 (0.52, 0.53) | 0.52 (0.51, 0.53) | 0.11 (-0.33, 0.56) | 681.35 | 0.23 |  |
|  |  |  | - | Male | 0.53 (0.52, 0.53) | 0.52 (0.51, 0.53) | 0.27 (-0.11, 0.65) | 690.90 | 0.19 |  |
|  |  | 90 | - | Female | 0.52 (0.51, 0.53) | 0.52 (0.51, 0.53) | -0.01 (-0.56, 0.53) | 1153.32 | 0.28 |  |
|  |  |  | - | Male | 0.53 (0.52, 0.53) | 0.52 (0.51, 0.53) | 0.08 (-0.41, 0.57) | 1255.96 | 0.25 |  |
|  |  | 180 | - | Female | 0.52 (0.51, 0.52) | 0.53 (0.52, 0.54) | -0.67 (-1.22, -0.12)* | 1154.70 | 0.28 |  |
|  |  |  | - | Male | 0.53 (0.52, 0.53) | 0.52 (0.51, 0.53) | 0.58 (0.11, 1.05)* | 1159.84 | 0.24 |  |
|  | Thalamus | 10 | - | Female | 0.53 (0.52, 0.54) | 0.53 (0.52, 0.54) | 0.01 (-0.46, 0.47) | 670.07 | 0.24 |  |
|  |  |  | - | Male | 0.53 (0.53, 0.54) | 0.53 (0.52, 0.53) | 0.23 (-0.17, 0.63) | 679.28 | 0.20 |  |
|  |  | 90 | - | Female | 0.53 (0.52, 0.53) | 0.53 (0.52, 0.54) | -0.14 (-0.71, 0.42) | 1123.02 | 0.29 |  |
|  |  |  | - | Male | 0.53 (0.52, 0.54) | 0.53 (0.52, 0.54) | -0.11 (-0.62, 0.39) | 1225.26 | 0.26 |  |
|  |  | 180 | - | Female | 0.52 (0.52, 0.53) | 0.54 (0.53, 0.55) | -0.77 (-1.34, -0.21)* | 1126.44 | 0.29 |  |
|  |  |  | - | Male | 0.54 (0.53, 0.54) | 0.53 (0.52, 0.54) | 0.42 (-0.06, 0.91) | 1131.21 | 0.25 |  |
|  | Amygdala | 10 | - | Female | 0.56 (0.56, 0.57) | 0.56 (0.55, 0.57) | 0.12 (-0.24, 0.47) | 764.69 | 0.18 |  |
|  |  |  | - | Male | 0.56 (0.56, 0.57) | 0.56 (0.55, 0.57) | 0.13 (-0.17, 0.44) | 774.39 | 0.15 |  |
|  |  | 90 | - | Female | 0.56 (0.56, 0.57) | 0.56 (0.55, 0.57) | 0.15 (-0.31, 0.61) | 1291.79 | 0.24 |  |
|  |  |  | - | Male | 0.56 (0.56, 0.57) | 0.56 (0.55, 0.57) | -0.06 (-0.48, 0.35) | 1381.85 | 0.21 |  |
|  |  | 180 | - | Female | 0.56 (0.55, 0.57) | 0.57 (0.56, 0.58) | -0.62 (-1.09, -0.16)* | 1280.05 | 0.24 |  |
|  |  |  | - | Male | 0.56 (0.56, 0.57) | 0.56 (0.55, 0.57) | 0.25 (-0.15, 0.65) | 1287.44 | 0.20 |  |
| *Note.* * = robust difference (i.e., *d* 95% CI $\neq$ 0). CI = confidence interval; SE = standard error; TBI = traumatic brain injury; OI = orthopedic injury. | | | | | | | | | |  |

***Supplemental Table 2* Follow-up pairwise comparisons for significant differences (FDR corrected *p* < .025) in regional (nodal) metrics among symptom groups based on child (top; see Fig. 3-4) and parent (bottom; see Fig. 5-6) report.** Pairwise comparisons conducted within the context of the final fitted model.

|  |  | |  | |  | |  | | ***Estimated Marginal Means (95% CI)*** | | | | | | | ***Mild TBI with persistent symptoms – TBI without persistent symptoms*** | | | | | | ***Mild TBI with persistent symptoms - OI*** | | | | | | ***Mild TBI without persistent symptoms - OI*** | | | | | | |
| --- | --- | --- | --- | --- | --- | --- | --- | --- | --- | --- | --- | --- | --- | --- | --- | --- | --- | --- | --- | --- | --- | --- | --- | --- | --- | --- | --- | --- | --- | --- | --- | --- | --- | --- |
| ***Metric*** | ***Region (node)*** | | ***Age at injury*** | | ***Sex*** | | ***Time (days) post-injury*** | | ***Mild TBI with persistent symptoms*** | | ***Mild TBI without persistent symptoms*** | | ***Mild OI*** | | ***Cohen’s d*** | | | ***df*** | | ***SE*** | | ***d*** | ***df*** | ***SE*** | | ***d*** | | | ***df*** | | ***SE*** | |  |  |
| **Child** | | | | | | | | | | | | | | | | | | | | | | | | | | | | | | | | | |  |
| Betweenness centrality | | | | | | | | | | | | | | | | | | | | | | | | | | | | | | | | | |  |
|  | Supramarginal gyrus | | | Younger  (10^th^ percentile) | | - | | 10 | | 1.37 (0.58, 2.17) | | 0.62 (0.14, 1.11) | | 0.67 (0.16, 1.18) | | | 0.28 (-0.07, 0.63) | | 964.08 | | 0.18 | 0.27 (-0.09, 0.62) | 965.60 | | 0.18 | | -0.02 (-0.28, 0.25) | | | 1001.06 | | 0.14 | |  |
|  |  | | |  | | - | | 90 | | -0.09 (-1.15, 0.98) | | 0.49 (-0.24, 1.21) | | 1.67 (0.85, 2.48) | | | -0.22 (-0.70, 0.27) | | 1033.69 | | 0.25 | -0.66 (-1.17, -0.16)* | 1057.52 | | 0.26 | | -0.45 (-0.86, -0.03)* | | | 1091.99 | | 0.21 | |  |
|  |  | | |  | | - | | 180 | | -0.29 (-1.66, 1.08) | | 0.63 (-0.03, 1.30) | | 0.28 (-0.45, 1.00) | | | -0.35 (-0.92, 0.23) | | 1097.54 | | 0.29 | -0.21 (-0.80, 0.37) | 1093.48 | | 0.30 | | 0.14 (-0.23, 0.51) | | | 1071.14 | | 0.19 | |  |
|  |  | | | Older  (90^th^ percentile) | | - | | 10 | | -0.05 (-0.91, 0.81) | | 0.57 (0.03, 1.11) | | 0.61 (0.07, 1.16) | | | -0.23 (-0.62, 0.15) | | 992.60 | | 0.20 | -0.25 (-0.63, 0.13) | 998.36 | | 0.20 | | -0.02 (-0.30, 0.27) | | | 962.72 | | 0.15 | |  |
|  |  | | |  | | - | | 90 | | 2.79 (1.67, 3.90) | | 0.48 (-0.37, 1.33) | | 0.43 (-0.33, 1.20) | | | 0.87 (0.34, 1.40)* | | 1079.86 | | 0.27 | 0.89 (0.38, 1.40)* | 1075.76 | | 0.26 | | 0.02 (-0.41, 0.45) | | | 1044.98 | | 0.22 | |  |
|  |  | | |  | | - | | 180 | | 0.62 (-0.61, 1.85) | | 0.48 (-0.30, 1.26) | | 0.85 (0.10, 1.61) | | | 0.05 (-0.50, 0.60) | | 1099.96 | | 0.28 | -0.09 (-0.63, 0.46) | 1112.78 | | 0.28 | | -0.14 (-0.55, 0.27) | | | 1107.79 | | 0.21 | |  |
| Efficiency | | | | | | | | | | | | | | | | | | | | | | | | | | | | | | | | | |  |
|  | | Putamen | | Younger | | - | | 10 | | 0.39 (0.38, 0.40) | | 0.39 (0.38, 0.39) | | 0.39 (0.38, 0.39) | | | 0.05 (-0.77, 0.88) | | 535.95 | | 0.42 | 0.03 (-0.81, 0.87) | 534.82 | | 0.43 | | -0.02 (-0.63, 0.58) | | | 552.15 | | 0.31 | |  |
|  | |  | |  | | - | | 90 | | 0.39 (0.38, 0.40) | | 0.39 (0.38, 0.39) | | 0.40 (0.39, 0.40) | | | 0.59 (-0.35, 1.53) | | 806.97 | | 0.48 | -0.49 (-1.46, 0.48) | 829.94 | | 0.49 | | -1.08 (-1.83, -0.33)* | | | 974.97 | | 0.38 | |  |
|  | |  | |  | | - | | 180 | | 0.39 (0.38, 0.40) | | 0.39 (0.38, 0.39) | | 0.39 (0.39, 0.40) | | | 0.61 (-0.42, 1.65) | | 995.12 | | 0.53 | 0.02 (-1.03, 1.07) | 995.04 | | 0.54 | | -0.59 (-1.30, 0.12) | | | 853.33 | | 0.36 | |  |
|  | |  | | Older | | - | | 10 | | 0.42 (0.41, 0.43) | | 0.42 (0.41, 0.42) | | 0.41 (0.41, 0.42) | | | 0.14 (-0.74, 1.03) | | 543.18 | | 0.45 | 0.80 (-0.08, 1.68) | 548.55 | | 0.45 | | 0.66 (-0.02, 1.34) | | | 526.80 | | 0.35 | |  |
|  | |  | |  | | - | | 90 | | 0.41 (0.40, 0.42) | | 0.42 (0.41, 0.43) | | 0.41 (0.40, 0.41) | | | -1.02 (-2.04, -0.01)* | | 819.8 | | 0.52 | 0.28 (-0.71, 1.27) | 787.24 | | 0.51 | | 1.30 (0.49, 2.11)* | | | 877.57 | | 0.41 | |  |
|  | |  | |  | | - | | 180 | | 0.41 (0.40, 0.42) | | 0.42 (0.41, 0.43) | | 0.41 (0.41, 0.42) | | | -0.71 (-1.74, 0.33) | | 861.42 | | 0.53 | -0.08 (-1.11, 0.95) | 855.98 | | 0.52 | | 0.63 (-0.15, 1.41) | | | 809.67 | | 0.40 | |  |
| Clustering coefficient | | | | | | | | | | | | | | | | | | | | | | | | | | | | | | | | | |  |
|  | | Rolandic operculum | | - | | Female | | - | | 0.59 (0.58, 0.60) | | 0.61 (0.60, 0.63) | | 0.60 (0.59, 0.61) | | | -0.89 (-1.49, -0.28)* | | 916.27 | | 0.31 | -0.53 (-1.08, 0.01) | 891.79 | | 0.28 | | 0.35 (-0.18, 0.89) | | | 1044.63 | | 0.27 | |  |
|  | |  | | - | | Male | | - | | 0.62 (0.61, 0.64) | | 0.60 (0.59, 0.61) | | 0.60 (0.59, 0.60) | | | 0.80 (0.19, 1.42)* | | 1013.7 | | 0.31 | 1.00 (0.36, 1.63)* | 1013.44 | | 0.32 | | 0.19 (-0.21, 0.60) | | | 1091.78 | | 0.21 | |  |
|  | | Thalamus | | - | | Female | | 10 | | 0.52 (0.51, 0.54) | | 0.54 (0.53, 0.55) | | 0.53 (0.52, 0.54) | | | -0.59 (-1.30, 0.12) | | 554.71 | | 0.36 | -0.24 (-0.90, 0.42) | 555.56 | | 0.34 | | 0.35 (-0.22, 0.92) | | | 552.74 | | 0.29 | |  |
|  | |  | | - | | Male | |  | | 0.54 (0.53, 0.55) | | 0.53 (0.53, 0.54) | | 0.53 (0.52, 0.53) | | | 0.38 (-0.31, 1.07) | | 535.87 | | 0.35 | 0.56 (-0.16, 1.27) | 541.33 | | 0.36 | | 0.17 (-0.26, 0.60) | | | 564.54 | | 0.22 | |  |
|  | |  | | - | | Female | | 90 | | 0.53 (0.52, 0.54) | | 0.54 (0.52, 0.55) | | 0.53 (0.52, 0.54) | | | -0.30 (-1.12, 0.51) | | 831.53 | | 0.42 | 0.00 (-0.75, 0.74) | 790.39 | | 0.38 | | 0.30 (-0.41, 1.02) | | | 986.02 | | 0.36 | |  |
|  | |  | | - | | Male | |  | | 0.53 (0.51, 0.55) | | 0.53 (0.52, 0.54) | | 0.53 (0.52, 0.54) | | | -0.04 (-0.87, 0.79) | | 912.22 | | 0.42 | -0.08 (-0.94, 0.78) | 922.48 | | 0.44 | | -0.04 (-0.59, 0.50) | | | 1031.38 | | 0.28 | |  |
|  | |  | | - | | Female | | 180 | | 0.50 (0.48, 0.52) | | 0.53 (0.52, 0.54) | | 0.54 (0.53, 0.55) | | | -1.54 (-2.55, -0.54)* | | 1204.01 | | 0.51 | -1.95 (-2.90, -1.01)* | 1220.48 | | 0.48 | | -0.41 (-1.09, 0.27) | | | 891.21 | | 0.35 | |  |
|  | |  | | - | | Male | |  | | 0.54 (0.53, 0.56) | | 0.54 (0.53, 0.54) | | 0.53 (0.52, 0.54) | | | 0.43 (-0.34, 1.20) | | 748.45 | | 0.39 | 0.78 (-0.03, 1.58) | 790.14 | | 0.41 | | 0.35 (-0.17, 0.87) | | | 916.23 | | 0.26 | |  |
|  | | Putamen | | - | | Female | | 10 | | 0.52 (0.50, 0.53) | | 0.53 (0.52, 0.54) | | 0.52 (0.51, 0.53) | | | -0.62 (-1.30, 0.06) | | 563.77 | | 0.35 | -0.17 (-0.80, 0.46) | 564.71 | | 0.32 | | 0.45 (-0.10, 1.00) | | | 561.86 | | 0.28 | |  |
|  | |  | | - | | Male | |  | | 0.53 (0.52, 0.55) | | 0.52 (0.52, 0.53) | | 0.52 (0.51, 0.53) | | | 0.46 (-0.20, 1.13) | | 543.36 | | 0.34 | 0.65 (-0.04, 1.33) | 549.23 | | 0.35 | | 0.18 (-0.23, 0.59) | | | 574.20 | | 0.21 | |  |
|  | |  | | - | | Female | | 90 | | 0.52 (0.51, 0.53) | | 0.53 (0.52, 0.55) | | 0.52 (0.51, 0.53) | | | -0.56 (-1.35, 0.23) | | 854.15 | | 0.40 | 0.01 (-0.71, 0.73) | 812.43 | | 0.37 | | 0.57 (-0.12, 1.27) | | | 1011.95 | | 0.35 | |  |
|  | |  | | - | | Male | |  | | 0.53 (0.51, 0.55) | | 0.53 (0.52, 0.53) | | 0.52 (0.51, 0.53) | | | 0.22 (-0.59, 1.02) | | 939.91 | | 0.41 | 0.30 (-0.53, 1.14) | 949.73 | | 0.42 | | 0.09 (-0.44, 0.62) | | | 1058.11 | | 0.27 | |  |
|  | |  | | - | | Female | | 180 | | 0.49 (0.47, 0.51) | | 0.52 (0.51, 0.53) | | 0.53 (0.52, 0.54) | | | -1.49 (-2.47, -0.51)* | | 1224.24 | | 0.50 | -1.82 (-2.74, -0.90)* | 1240.14 | | 0.47 | | -0.33 (-0.99, 0.33) | | | 915.82 | | 0.34 | |  |
|  | |  | | - | | Male | |  | | 0.54 (0.52, 0.55) | | 0.53 (0.52, 0.53) | | 0.52 (0.51, 0.53) | | | 0.50 (-0.24, 1.24) | | 770.65 | | 0.38 | 0.98 (0.20, 1.76)* | 813.42 | | 0.4 | | 0.48 (-0.02, 0.98) | | | 940.58 | | 0.26 | |  |
|  | | Amygdala | | - | | Female | | 10 | | 0.56 (0.55, 0.58) | | 0.57 (0.56, 0.58) | | 0.56 (0.55, 0.57) | | | -0.11 (-0.67, 0.45) | | 617.64 | | 0.28 | 0.11 (-0.41, 0.63) | 618.99 | | 0.27 | | 0.22 (-0.23, 0.67) | | | 616.70 | | 0.23 | |  |
|  | |  | | - | | Male | |  | | 0.57 (0.55, 0.58) | | 0.56 (0.56, 0.57) | | 0.56 (0.55, 0.57) | | | 0.22 (-0.32, 0.76) | | 588.79 | | 0.28 | 0.30 (-0.26, 0.86) | 596.76 | | 0.28 | | 0.08 (-0.26, 0.42) | | | 630.52 | | 0.17 | |  |
|  | |  | | - | | Female | | 90 | | 0.57 (0.56, 0.58) | | 0.57 (0.56, 0.58) | | 0.56 (0.55, 0.57) | | | -0.12 (-0.80, 0.56) | | 952.73 | | 0.35 | 0.39 (-0.22, 1.01) | 914.16 | | 0.31 | | 0.51 (-0.10, 1.12) | | | 1110.40 | | 0.31 | |  |
|  | |  | | - | | Male | |  | | 0.56 (0.54, 0.57) | | 0.56 (0.56, 0.57) | | 0.56 (0.55, 0.57) | | | -0.22 (-0.92, 0.48) | | 1057.9 | | 0.36 | -0.21 (-0.94, 0.51) | 1063.83 | | 0.37 | | 0.01 (-0.46, 0.47) | | | 1156.39 | | 0.24 | |  |
|  | |  | | - | | Female | | 180 | | 0.53 (0.51, 0.55) | | 0.56 (0.55, 0.57) | | 0.57 (0.56, 0.58) | | | -1.14 (-2.02, -0.27)* | | 1279.44 | | 0.45 | -1.66 (-2.48, -0.83)* | 1292.14 | | 0.42 | | -0.51 (-1.09, 0.06) | | | 1019.24 | | 0.29 | |  |
|  | |  | | - | | Male | |  | | 0.57 (0.56, 0.58) | | 0.56 (0.56, 0.57) | | 0.56 (0.55, 0.57) | | | 0.26 (-0.37, 0.89) | | 881.88 | | 0.32 | 0.47 (-0.20, 1.13) | 924.60 | | 0.34 | | 0.21 (-0.23, 0.64) | | | 1039.64 | | 0.22 | |  |
| **Parent** | | | | | | | | | | | | | | | | | | | | | | | | | | | | | | | | | |  |
| Clustering coefficient | | | | | | | | | | | | | | | | | | | | | | | | | | | | | | | | | |  |
|  | | Middle occipital gyrus | | - | | - | | 10 | | 0.58 (0.57, 0.59) | | 0.59 (0.59, 0.60) | | 0.58 (0.58, 0.59) | | | -0.45 (-0.81, -0.10)* | | 656.74 | | 0.18 | -0.11 (-0.46, 0.25) | 659.52 | | 0.18 | | 0.35 (0.11, 0.58)* | | | 699.34 | | 0.12 | |  |
|  | |  | | - | | - | | 90 | | 0.59 (0.57, 0.60) | | 0.59 (0.58, 0.60) | | 0.58 (0.57, 0.59) | | | -0.11 (-0.58, 0.36) | | 1124.16 | | 0.24 | 0.12 (-0.36, 0.61) | 1141.23 | | 0.25 | | 0.23 (-0.09, 0.56) | | | 1165.46 | | 0.17 | |  |
|  | |  | | - | | - | | 180 | | 0.57 (0.56, 0.59) | | 0.58 (0.58, 0.59) | | 0.59 (0.58, 0.59) | | | -0.38 (-0.90, 0.15) | | 1237.21 | | 0.27 | -0.49 (-1.01, 0.04) | 1231.42 | | 0.27 | | -0.11 (-0.43, 0.21) | | | 1126.24 | | 0.16 | |  |
|  | | Hippocampus | | - | | - | | 10 | | 0.54 (0.53, 0.55) | | 0.55 (0.54, 0.55) | | 0.54 (0.54, 0.55) | | | -0.14 (-0.63, 0.34) | | 563.58 | | 0.25 | 0.01 (-0.47, 0.50) | 565.26 | | 0.25 | | 0.15 (-0.17, 0.47) | | | 589.38 | | 0.16 | |  |
|  | |  | | - | | - | | 90 | | 0.54 (0.53, 0.56) | | 0.55 (0.54, 0.55) | | 0.54 (0.54, 0.55) | | | -0.08 (-0.68, 0.51) | | 963.47 | | 0.30 | 0.05 (-0.55, 0.65) | 982.72 | | 0.30 | | 0.14 (-0.26, 0.54) | | | 1031.19 | | 0.20 | |  |
|  | |  | | - | | - | | 180 | | 0.53 (0.52, 0.55) | | 0.54 (0.54, 0.55) | | 0.55 (0.54, 0.55) | | | -0.44 (-1.08, 0.20) | | 1118.78 | | 0.33 | -0.73 (-1.37, -0.09)* | 1108.16 | | 0.33 | | -0.29 (-0.68, 0.11) | | | 994.14 | | 0.20 | |  |
|  | | Caudate | | - | | - | | 10 | | 0.52 (0.51, 0.53) | | 0.52 (0.51, 0.52) | | 0.52 (0.51, 0.52) | | | -0.13 (-0.63, 0.37) | | 556.51 | | 0.26 | 0.08 (-0.43, 0.58) | 558.08 | | 0.26 | | 0.20 (-0.13, 0.54) | | | 580.66 | | 0.17 | |  |
|  | |  | | - | | - | | 90 | | 0.52 (0.51, 0.54) | | 0.52 (0.51, 0.53) | | 0.52 (0.51, 0.52) | | | 0.19 (-0.41, 0.80) | | 940.21 | | 0.31 | 0.40 (-0.21, 1.02) | 959.21 | | 0.31 | | 0.21 (-0.20, 0.62) | | | 1008.98 | | 0.21 | |  |
|  | |  | | - | | - | | 180 | | 0.51 (0.49, 0.52) | | 0.52 (0.51, 0.53) | | 0.52 (0.51, 0.52) | | | -0.67 (-1.33, -0.01)* | | 1096.29 | | 0.34 | -0.58 (-1.23, 0.08) | 1085.37 | | 0.33 | | 0.09 (-0.31, 0.50) | | | 973.33 | | 0.21 | |  |
|  | | Amygdala | | - | | - | | 10 | | 0.56 (0.55, 0.57) | | 0.57 (0.56, 0.57) | | 0.56 (0.56, 0.57) | | | -0.12 (-0.52, 0.29) | | 609.09 | | 0.21 | 0.06 (-0.35, 0.46) | 611.36 | | 0.21 | | 0.17 (-0.09, 0.44) | | | 644.25 | | 0.14 | |  |
|  | |  | | - | | - | | 90 | | 0.57 (0.56, 0.58) | | 0.56 (0.56, 0.57) | | 0.56 (0.55, 0.57) | | | 0.28 (-0.24, 0.80) | | 1069.70 | | 0.26 | 0.35 (-0.17, 0.88) | 1088.56 | | 0.27 | | 0.08 (-0.28, 0.43) | | | 1124.75 | | 0.18 | |  |
|  | |  | | - | | - | | 180 | | 0.55 (0.53, 0.56) | | 0.56 (0.56, 0.57) | | 0.57 (0.56, 0.57) | | | -0.65 (-1.23, -0.08)* | | 1206.66 | | 0.29 | -0.78 (-1.35, -0.21)* | 1198.50 | | 0.29 | | -0.13 (-0.47, 0.22) | | | 1083.71 | | 0.18 | |  |
|  | | Superior parietal gyrus | | - | | Female | | 10 | | 0.56 (0.55, 0.57) | | 0.58 (0.57, 0.58) | | 0.57 (0.56, 0.58) | | | -0.64 (-1.22, -0.07)* | | 639.30 | | 0.29 | -0.48 (-1.04, 0.08) | 636.93 | | 0.29 | | 0.17 (-0.23, 0.56) | | | 667.48 | | 0.20 | |  |
|  | |  | | - | | Male | |  | | 0.57 (0.56, 0.58) | | 0.57 (0.56, 0.58) | | 0.57 (0.56, 0.57) | | | 0.02 (-0.47, 0.50) | | 618.36 | | 0.25 | 0.06 (-0.44, 0.57) | 627.68 | | 0.26 | | 0.04 (-0.27, 0.36) | | | 679.03 | | 0.16 | |  |
|  | |  | | - | | Female | | 90 | | 0.57 (0.56, 0.59) | | 0.57 (0.56, 0.58) | | 0.58 (0.57, 0.59) | | | 0.02 (-0.69, 0.73) | | 1013.47 | | 0.36 | -0.23 (-0.93, 0.48) | 1039.73 | | 0.36 | | -0.25 (-0.76, 0.27) | | | 1105.41 | | 0.26 | |  |
|  | |  | | - | | Male | |  | | 0.57 (0.55, 0.59) | | 0.57 (0.57, 0.58) | | 0.57 (0.56, 0.58) | | | -0.18 (-0.85, 0.50) | | 1186.12 | | 0.34 | 0.00 (-0.71, 0.70) | 1187.61 | | 0.36 | | 0.18 (-0.26, 0.61) | | | 1204.41 | | 0.22 | |  |
|  | |  | | - | | Female | | 180 | | 0.53 (0.50, 0.55) | | 0.57 (0.56, 0.58) | | 0.58 (0.57, 0.59) | | | -1.60 (-2.45, -0.74)* | | 1258.23 | | 0.44 | -1.85 (-2.67, -1.03)* | 1243.22 | | 0.42 | | -0.25 (-0.78, 0.27) | | | 1116.02 | | 0.27 | |  |
|  | |  | | - | | Male | |  | | 0.57 (0.56, 0.59) | | 0.57 (0.56, 0.58) | | 0.56 (0.55, 0.57) | | | 0.12 (-0.56, 0.79) | | 1185.44 | | 0.34 | 0.41 (-0.30, 1.12) | 1195.47 | | 0.36 | | 0.29 (-0.12, 0.70) | | | 1103.70 | | 0.21 | |  |
|  | | Precuneus | | - | | Female | | 10 | | 0.55 (0.54, 0.57) | | 0.56 (0.55, 0.57) | | 0.55 (0.55, 0.56) | | | -0.21 (-0.87, 0.45) | | 595.26 | | 0.34 | -0.01 (-0.66, 0.63) | 593.14 | | 0.33 | | 0.20 (-0.25, 0.65) | | | 616.70 | | 0.23 | |  |
|  | |  | | - | | Male | |  | | 0.57 (0.55, 0.58) | | 0.56 (0.56, 0.57) | | 0.56 (0.55, 0.56) | | | 0.19 (-0.38, 0.75) | | 577.41 | | 0.29 | 0.35 (-0.23, 0.94) | 585.21 | | 0.30 | | 0.17 (-0.19, 0.53) | | | 628.51 | | 0.18 | |  |
|  | |  | | - | | Female | | 90 | | 0.56 (0.54, 0.58) | | 0.56 (0.55, 0.57) | | 0.56 (0.55, 0.57) | | | 0.07 (-0.72, 0.86) | | 941.31 | | 0.40 | 0.06 (-0.72, 0.85) | 965.13 | | 0.40 | | 0.00 (-0.57, 0.56) | | | 1044.73 | | 0.29 | |  |
|  | |  | | - | | Male | |  | | 0.56 (0.55, 0.58) | | 0.56 (0.55, 0.57) | | 0.55 (0.54, 0.56) | | | 0.16 (-0.58, 0.90) | | 1117.81 | | 0.38 | 0.51 (-0.26, 1.27) | 1123.05 | | 0.39 | | 0.35 (-0.13, 0.82) | | | 1154.29 | | 0.24 | |  |
|  | |  | | - | | Female | | 180 | | 0.52 (0.50, 0.54) | | 0.55 (0.54, 0.56) | | 0.57 (0.56, 0.58) | | | -1.07 (-2.01, -0.14)* | | 1217.03 | | 0.48 | -1.83 (-2.72, -0.93)* | 1195.15 | | 0.46 | | -0.75 (-1.33, -0.18)* | | | 1061.59 | | 0.29 | |  |
|  | |  | | - | | Male | |  | | 0.57 (0.55, 0.59) | | 0.56 (0.55, 0.57) | | 0.55 (0.54, 0.56) | | | 0.41 (-0.33, 1.15) | | 1115.68 | | 0.38 | 0.73 (-0.05, 1.50) | 1131.53 | | 0.40 | | 0.32 (-0.13, 0.77) | | | 1045.02 | | 0.23 | |  |
|  | | Thalamus | | - | | Female | | 10 | | 0.52 (0.51, 0.54) | | 0.53 (0.53, 0.54) | | 0.53 (0.52, 0.54) | | | -0.57 (-1.37, 0.23) | | 552.73 | | 0.41 | -0.36 (-1.14, 0.43) | 551.08 | | 0.40 | | 0.22 (-0.33, 0.76) | | | 567.18 | | 0.28 | |  |
|  | |  | | - | | Male | |  | | 0.53 (0.52, 0.55) | | 0.53 (0.53, 0.54) | | 0.53 (0.52, 0.53) | | | 0.11 (-0.58, 0.79) | | 539.54 | | 0.35 | 0.27 (-0.44, 0.98) | 545.32 | | 0.36 | | 0.17 (-0.27, 0.60) | | | 577.28 | | 0.22 | |  |
|  | |  | | - | | Female | | 90 | | 0.53 (0.52, 0.55) | | 0.53 (0.52, 0.54) | | 0.53 (0.52, 0.54) | | | 0.07 (-0.85, 0.99) | | 830.72 | | 0.47 | 0.10 (-0.80, 1.01) | 849.28 | | 0.46 | | 0.03 (-0.62, 0.68) | | | 933.07 | | 0.33 | |  |
|  | |  | | - | | Male | |  | | 0.53 (0.51, 0.54) | | 0.53 (0.52, 0.54) | | 0.53 (0.52, 0.54) | | | -0.04 (-0.89, 0.80) | | 987.11 | | 0.43 | -0.15 (-1.03, 0.73) | 996.29 | | 0.45 | | -0.11 (-0.65, 0.43) | | | 1042.39 | | 0.28 | |  |
|  | |  | | - | | Female | | 180 | | 0.49 (0.47, 0.51) | | 0.53 (0.52, 0.54) | | 0.54 (0.53, 0.55) | | | -1.97 (-3.02, -0.91)* | | 1110.17 | | 0.54 | -2.42 (-3.44, -1.40)* | 1081.13 | | 0.52 | | -0.46 (-1.12, 0.21) | | | 955.87 | | 0.34 | |  |
|  | |  | | - | | Male | |  | | 0.54 (0.53, 0.56) | | 0.53 (0.53, 0.54) | | 0.53 (0.52, 0.54) | | | 0.41 (-0.43, 1.26) | | 985.45 | | 0.43 | 0.72 (-0.16, 1.61) | 1006.42 | | 0.45 | | 0.31 (-0.21, 0.83) | | | 937.05 | | 0.26 | |  |
|  | | Putamen | | - | | Female | | 10 | | 0.52 (0.50, 0.53) | | 0.53 (0.52, 0.53) | | 0.52 (0.51, 0.53) | | | -0.51 (-1.27, 0.25) | | 561.91 | | 0.39 | -0.19 (-0.94, 0.56) | 560.14 | | 0.38 | | 0.32 (-0.20, 0.83) | | | 577.90 | | 0.26 | |  |
|  | |  | | - | | Male | |  | | 0.53 (0.51, 0.54) | | 0.52 (0.52, 0.53) | | 0.52 (0.51, 0.53) | | | 0.14 (-0.51, 0.79) | | 547.58 | | 0.33 | 0.34 (-0.34, 1.01) | 553.84 | | 0.34 | | 0.20 (-0.22, 0.61) | | | 588.55 | | 0.21 | |  |
|  | |  | | - | | Female | | 90 | | 0.52 (0.51, 0.54) | | 0.53 (0.52, 0.54) | | 0.52 (0.51, 0.53) | | | -0.09 (-0.97, 0.80) | | 858.65 | | 0.45 | 0.09 (-0.78, 0.97) | 878.65 | | 0.45 | | 0.18 (-0.45, 0.81) | | | 963.07 | | 0.32 | |  |
|  | |  | | - | | Male | |  | | 0.53 (0.51, 0.54) | | 0.52 (0.52, 0.53) | | 0.52 (0.51, 0.53) | | | 0.23 (-0.58, 1.05) | | 1022.32 | | 0.42 | 0.27 (-0.58, 1.11) | 1030.76 | | 0.43 | | 0.03 (-0.49, 0.56) | | | 1074.16 | | 0.27 | |  |
|  | |  | | - | | Female | | 180 | | 0.49 (0.47, 0.51) | | 0.52 (0.51, 0.53) | | 0.53 (0.52, 0.54) | | | -1.60 (-2.62, -0.58)* | | 1141.70 | | 0.52 | -1.99 (-2.97, -1.01)* | 1113.99 | | 0.5 | | -0.39 (-1.03, 0.25) | | | 984.87 | | 0.33 | |  |
|  | |  | | - | | Male | |  | | 0.53 (0.52, 0.55) | | 0.53 (0.52, 0.53) | | 0.52 (0.51, 0.53) | | | 0.36 (-0.46, 1.17) | | 1020.41 | | 0.42 | 0.80 (-0.06, 1.65) | 1040.57 | | 0.44 | | 0.44 (-0.06, 0.94) | | | 966.20 | | 0.25 | |  |
|  | | Pallidum | | - | | Female | | 10 | | 0.54 (0.52, 0.55) | | 0.55 (0.54, 0.56) | | 0.54 (0.54, 0.55) | | | -0.49 (-1.21, 0.23) | | 574.18 | | 0.37 | -0.15 (-0.85, 0.56) | 572.26 | | 0.36 | | 0.34 (-0.15, 0.83) | | | 592.20 | | 0.25 | |  |
|  | |  | | - | | Male | |  | | 0.55 (0.54, 0.56) | | 0.55 (0.54, 0.55) | | 0.54 (0.54, 0.55) | | | 0.21 (-0.41, 0.82) | | 558.44 | | 0.31 | 0.26 (-0.38, 0.89) | 565.3 | | 0.32 | | 0.05 (-0.34, 0.44) | | | 603.43 | | 0.20 | |  |
|  | |  | | - | | Female | | 90 | | 0.55 (0.53, 0.57) | | 0.55 (0.54, 0.56) | | 0.54 (0.53, 0.55) | | | 0.10 (-0.75, 0.94) | | 892.34 | | 0.43 | 0.30 (-0.54, 1.13) | 913.99 | | 0.43 | | 0.20 (-0.40, 0.80) | | | 997.70 | | 0.31 | |  |
|  | |  | | - | | Male | |  | | 0.54 (0.53, 0.56) | | 0.55 (0.54, 0.56) | | 0.55 (0.54, 0.56) | | | -0.19 (-0.97, 0.59) | | 1062.97 | | 0.40 | -0.11 (-0.92, 0.71) | 1070.27 | | 0.42 | | 0.08 (-0.42, 0.59) | | | 1109.4 | | 0.26 | |  |
|  | |  | | - | | Female | | 180 | | 0.51 (0.48, 0.53) | | 0.55 (0.54, 0.56) | | 0.55 (0.54, 0.56) | | | -1.99 (-2.97, -1.01)* | | 1175.69 | | 0.50 | -1.95 (-2.89, -1.00)* | 1150.06 | | 0.48 | | 0.04 (-0.57, 0.65) | | | 1017.83 | | 0.31 | |  |
|  | |  | | - | | Male | |  | | 0.56 (0.54, 0.58) | | 0.55 (0.54, 0.55) | | 0.54 (0.53, 0.55) | | | 0.60 (-0.18, 1.39) | | 1060.84 | | 0.40 | 0.85 (0.03, 1.67)* | 1079.57 | | 0.42 | | 0.24 (-0.23, 0.72) | | | 999.71 | | 0.24 | |  |
| *Note.* * = robust difference (i.e., *d* 95% CI $\neq$ 0). CI = confidence interval; SE = standard error; TBI = traumatic brain injury; OI = orthopedic injury. | | | | | | | | | | | | | | | | | | | | | | | | | | | | | | | | | |  |
